# Fast wide-volume functional imaging of engineered *in vitro* brain tissues

**DOI:** 10.1038/s41598-017-08979-8

**Published:** 2017-08-17

**Authors:** G. Palazzolo, M. Moroni, A. Soloperto, G. Aletti, G. Naldi, M. Vassalli, T. Nieus, F. Difato

**Affiliations:** 1 Department of Neuroscience and Brain Technologies, Fondazione Istituto Italiano di Tecnologia, Genoa, Italy; 2 Center for Neuroscience and Cognitive Systems @UniTn, Istituto Italiano di Tecnologia, Rovereto, Italy; 3 Center for Mind/Brain Sciences, University of Trento, Rovereto, Italy; 4 Dipartimento di Matematica, Università degli studi di Milano, Milano, Italy; 5 Institute of Biophysics, National Research Council of Italy, Genoa, Italy; 6 Department of Biomedical and Clinical Sciences “L. Sacco”, Università degli Studi di Milano, Milano, Italy

## Abstract

The need for *in vitro* models that mimic the human brain to replace animal testing and allow high-throughput screening has driven scientists to develop new tools that reproduce tissue-like features on a chip. Three-dimensional (3D) *in vitro* cultures are emerging as an unmatched platform that preserves the complexity of cell-to-cell connections within a tissue, improves cell survival, and boosts neuronal differentiation. In this context, new and flexible imaging approaches are required to monitor the functional states of 3D networks. Herein, we propose an experimental model based on 3D neuronal networks in an alginate hydrogel, a tunable wide-volume imaging approach, and an efficient denoising algorithm to resolve, down to single cell resolution, the 3D activity of hundreds of neurons expressing the calcium sensor GCaMP6s. Furthermore, we implemented a 3D co-culture system mimicking the contiguous interfaces of distinct brain tissues such as the cortical-hippocampal interface. The analysis of the network activity of single and layered neuronal co-cultures revealed cell-type-specific activities and an organization of neuronal subpopulations that changed in the two culture configurations. Overall, our experimental platform represents a simple, powerful and cost-effective platform for developing and monitoring living 3D layered brain tissue on chip structures with high resolution and high throughput.

## Introduction

The design and development of realistic experimental models of the human brain remain major constraints in studying nervous system functions in health and disease, developing new treatment strategies, and testing prosthetic devices^1^. For these reasons, *in vitro* models represent an attractive and ethical tool since they allow high-throughput cost-accessible investigation^2^. Moreover, the development of cell reprogramming and differentiation protocols that allow investigators to recapitulate human diseases in a dish further discourages the use of animal models. The most common approach to building a brain on a chip is based on 2D cultures of defined neural cell types with a controlled network topology^3^. These models are highly reproducible and are compatible with standard monitoring systems^4^. However, growth of cells on a 2D support alters cellular physiology^5^ and fails to reproduce the real properties of a tissue. Growth on stiff Petri dish surfaces induces aberrant branching of neuronal processes^6^, which, together with the lower degree of freedom of neurite navigation in a 2D setting, typically promotes overloading of interneuronal connections. This, in turn, may cause the observed amplification of the activity of 2D neuronal networks on stiff substrates^7^, which may lead to non-physiological states^8^. In this regard, it is widely recognized that mechanical and topographical cues influence cell survival, proliferation and differentiation and finally the development of functional neural networks^9^. Indeed, the architecture of a 3D scaffold allows isotropic distribution of adhesion contacts, thereby modifying the intracellular distribution of mechanical tension and consequently nuclear shape, chromatin arrangement and gene expression in cells^10^. Moreover, mechanotaxis influences the motility of neuronal processes^6^, and it has a critical role in axonal pathfinding *in vivo* ^11^. For all of these reasons, there has been a growing demand for new technologies and materials (either natural or synthetic) to make it possible to produce 3D culture models that better resemble the topographical arrangement and the extracellular milieu of real tissues^12^.

At present, 3D cultures prepared in biocompatible hydrogels hold promise for re-creating small brains *in vitro*. Distinct approaches, including bioprinting^13^, 3D micropatterning^14^, the use of multi-composite materials^12^, and post-assembly of hydrogel tissue blocks^15^ have been proposed to recapitulate the functional compartmentalization of the brain *in vitro*. Mammalian-derived polymers are generally used to preserve the biochemical signatures of the native tissue. However, these polymers are easily recognized by cellular enzymes and thus often undergo rapid degradation. As an alternative, non-mammalian polymers can also promote cell differentiation and neurite extension^16^ while providing greater long-term stability^17^. However, the growth of *in vitro* neural networks in the third dimension has posed serious technological challenges in resolving the network circuitry over large volumes with adequate temporal and spatial resolution^18^. Optical approaches are the most exploited tools for correlating the structural and functional features of living neural networks. Novel optical systems based on ultrashort pulse lasers^19^ in combination with ultrafast^20^ and multiplexed^21^ scanning approaches or with adaptive light wavefront engineering and structured illumination methods^22, 23^ have significantly improved the penetration depth and the sampling speed that can be used in monitoring 3D neural networks. Moreover, new genetically encoded probes allow long-term, high-speed monitoring of the activity of such neural networks at remarkable signal-to-noise ratios^24^. Current state-of-the-art optical approaches use patterned illumination and parallel detection^23^, ultrafast sequential scanning and detection, or integration of parallel^25^ and sequential approaches with deconvolution and demixing algorithms^26^. However, most of them allow a few optical sections or locations to be monitored or provide a small field/volume of view because they require high numerical aperture objectives^18^.

Current approaches to measuring the correlation of the activity of distinct neuronal subpopulations located in distinct sample planes include fixed-phase mask modulation of the detection pathway to extend the microscopic depth of field^27^, phase modulation in the pupil plane of the microscope objective^28^, axial scanning of a sample using fast motorized elements^29^, and optical point spread function engineering to accomplish galvo-mirror-based volume scanning^30, 31^. To date, systems based on acoustic optical deflectors have achieved a breakthrough in terms of the volume and number of cells monitored per second^20^.

In the present work, we propose a simple strategy to produce 3D neuronal cultures and layered co-cultures in hydrogels consisting of the natural polysaccharide alginate. The great potential of alginate for building 3D cell cultures resides in that a) it is inexpensive and biocompatible; b) it is not recognized by mammalian cellular enzymes; c) the elasticity of alginate hydrogels can be tuned to match the range of stiffness of brain tissues^17^; and d) the optical transparency of alginate hydrogel favors the exploitation of optical methods^32^. We engineered 3D networks of cortical or hippocampal neurons and layered 3D networks of cortical and hippocampal co-cultures that express the genetically encoded calcium sensor GCaMP6s. We characterized the 3D physiological activity of these cultured cellular networks down to single-cell resolution over a long-term temporal window (up to two months), using a novel optical system to perform calcium imaging. The optical system was based on a simple wide-field architecture that was extended to a wide-volume imaging approach through controllable phase modulation of the detection pathway. We were able to monitor the activity of hundreds of neurons in a wide volume of 800 × 800 × 300 µm. The temporal resolution of our system in monitoring neuronal activity was limited only by the sensitivity of the detector; with the CCD detector used in this study, we obtain a wide-volume scanning rate of 65 Hz. Therefore, we achieved the current state of the art of 3D functional imaging^20, 33^ in terms of scanned volume extension and neuronal sampling rate^15^: we are able to monitor 20000 ÷ 35000 cells per second in a volume of ~0.19 mm^3^ using a simple and inertia-free optical architecture. The system was further equipped with an automated analysis platform to identify the 3D spatial localization of the cells and to detect calcium fluctuation events in the cells through a novel and highly efficient denoising algorithm to reconstruct and quantify the functional activity at both the single-cell and network levels. Overall, we present a reliable 3D *in vitro* experimental model of brain tissue that provides high-resolution concomitant structural and functional characterization and an experimental protocol for engineering 3D brain tissue interfaces on a chip^34^. Moreover, the whole model is easily accessible by any laboratories and is thus a versatile and efficient model for high-throughput screening studies.

## Results

### Engineered 3D neuronal networks in alginate hydrogels

**Figure 1:**
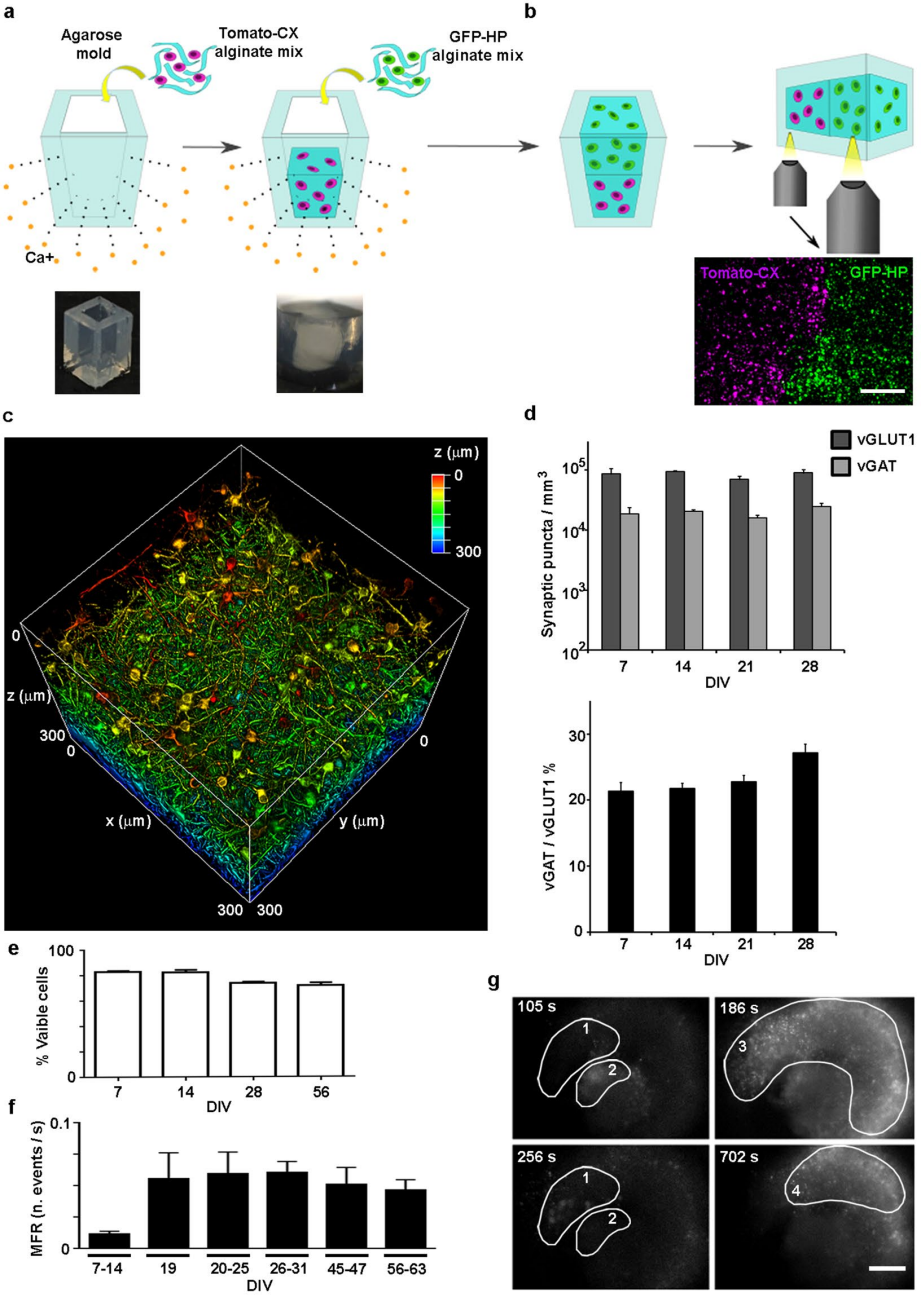
Engineering the 3D neural network and characterizing the synaptic markers and the long-term calcium activity. (**a**) Scheme of the procedure to build, within an agarose rectangular parallelepiped, a 3D culture of primary neurons embedded in an alginate hydrogel. Gelation occurred due to the flux of calcium ions through the matrix. Below from left to right, the photographs of the agarose mold and of the alginate hydrogel - obtained with the BLIPS MacroLens - are shown; (**b**) the same gelling procedure, repeated in two steps, allowed to obtain a 3D layered co-culture of td-Tomato-expressing cortical neurons and GFP-expressing hippocampal neurons. By horizontally flipping the mold, it was possible to image both the layers and the interface. Below, the maximum intensity projection of 160 μm thick z-stack of a layered co-culture at 4 DIVs, acquired with an inverted Leica SP5 confocal microscope (5x air Olympus objective), is shown; magenta: cortical neurons, green: hippocampal neurons. Bar is 500 µm (**c**) 3D reconstruction of 300 μm z-stack of a cortical culture at 53 DIVs (25x, NA1, water immersion Olympus objective) with depth color code is shown (supplementary Video S1); (**d**) Bar plots showing the numbers (bottom) of the excitatory (vGLUT1) and inhibitory (vGAT) synaptic puncta per mm^3^t (top), and the vGAT/vGLUT1% ratio (bottom) We quantified the number of vGAT and vGLUT1 synaptic puncta per mm^3^ on three different confocal z-stacks (volume 310 × 310 × 20 µm) acquired on each of the three distinct hydrogels (n = 3) analyzed for each group of DIVs reported; data are shown as mean values + standard errors; (**e**) Bar plot of the percent of viable cells of 3D cortical cultures over time. We count an average value of about 140 ± 36 cells nuclei on a total of 36 confocal z-stacks with a volume of 310 × 310 × 40 µm. In details, we analyzed three distinct z-stacks for 3 distinct hydrogels, in each reported condition; (**f**) Bar plot showing the mean firing rate (MFR), expressed as number of events per second, of 3D cortical cultures over time; data shown are the mean values + standard errors, (n = 6) for each DIVs interval; (**g**) Four frames of fluorescence calcium imaging acquired on a GCaMP6s-expressing 3D cortical culture at 26 DIVs. The frames are extracted from a time lapse movie acquired with a wide field microscope equipped with a 4x air Olympus objective (supplementary video S2). Frame rate 20 Hz, FOV 2.4 × 1.8 mm. The images illustrate four distinct spatial patterns of activity, which are highlighted with numbered white circles. On the upper left corner, it is reported the time of the events. Bar is 350 µm.

*In vitro* 3D models of neuronal networks must provide realistic 3D functional connectivity and easy access to imaging tools for an efficient and reliable readout of neuronal activity. To fulfill these features, we engineered a rectangular parallelepiped-shaped agarose mold (Fig. 1a, bottom left) into which a neuron-alginate mixture could be loaded to form a hydrogel due to the flux of calcium ions throughout the agarose (Fig. 1a). The slow gelation of the polymer containing the cell mixture resulted in a homogeneous 3D hydrogel approximately 2 mm thick with an opacity and elasticity^17^ similar to those of native brain tissue (Fig. 1a, bottom right). In Fig. 1c, we show a representative 3D reconstruction illustrating the distribution of cortical neurons within the hydrogel. Neurons were labeled with βIII-tubulin, which is well distributed throughout the cell soma and neurites. Figure 1c and the supplementary Video S1 illustrate the complexity of the network and the effective volumetric distribution of the cells and neuronal connections within the scaffold. Moreover, the novel mold structure allowed us to incrementally build a layered co-culture and to easily visualize the contiguous interface between the two distinct neuronal networks. As an example, we report in Fig. 1b the design of a cortical hippocampal tissue interface in which the bottom layer consists of alginate pre-mixed with primary cortical neurons expressing the td-Tomato protein and the top layer consists of primary hippocampal neurons expressing the GFP protein. The rectangular parallelepiped-shaped agar mold allows the easy manipulation and reorientation of the neuronal culture, thus providing easy optical access to the distinct faces of the culture while minimizing spherical optical aberrations induced by the cylindrical agar mold shape that was previously used^17^. For example, horizontal flipping of the agarose mold after gelation allowed easy visualization of the contiguous interface between the neuronal networks consisting of distinct cell types (Fig. 1b, bottom). Thus, our system represents an optically accessible *in vitro* model of the layered structure of the brain at the hippocampal-cortical interface.

To characterize the physiological relevance of the 3D neuronal network model, we analyzed the presence and localization of excitatory and inhibitory synaptic puncta during the first month of neural network development by immunostaining of the markers vGLUT1 and vGAT. Figure 1d (top) shows a quantification of the number of vGAT and vGLUT1 synaptic puncta per mm^3^ computed on three different confocal z-stacks (the volume of the z-stack was 310 × 310 × 20 µm) acquired on each of the three distinct hydrogels (n = 3) analyzed at the reported time points. The lower panel of Fig. 1d shows the vGAT/vGLUT1 ratio, which was maintained at approximately 22% from 7 days *in vitro* (DIVs) to 21 DIVs; it became significantly higher at 28 DIVs (27% ± 1.2; 7 vs 28 DIVs p = 0.018; 14 vs 28 DIVs p = 0.036; 21 vs 28 DIVs p = 0.14). Moreover, we observed that whereas vGLUT1 and vGAT localized along different dendrites at 7 DIVs, at 28 DIVs the two markers localized along the same dendrites and the cell somata (supplementary Fig. S1), indicating that a process of neuronal maturation similar to that occurring *in vivo* took place^35^.

From previously reported values of vGLUT1 and vGAT puncta densities in the layers of the rat somatosensory cortex^36^, we estimated the *in vivo* vGLUT1 and vGAT densities to be in the range of 1.6 ÷ 11.5 × 10^7^ and 1.5 ÷ 8 × 10^7^ puncta per mm^3^, respectively. Considering the estimated cell density of 1.2 × 10^6^ per mm^3^, which was confirmed in three distinct areas of the rat cortex^37^, we calculated that the distinct layers of the rat cortex had a range of approximately 13 ÷ 95 vGLUT1 synaptic puncta and 12 ÷ 66 vGAT synaptic puncta per cell. In our *in vitro* 3D model, we observed a lower cell density (~4 × 10^3^ cells per mm^3^), corresponding to a density of vGLUT1 and vGAT synaptic puncta per cell of approximately 25 ± 5 and 6 ± 1.5, respectively. These numbers indicated that although the cell density of our 3D neuronal networks was lower than that of the real tissue, the number of synaptic puncta per neuron was comparable.

To further confirm the physiological relevance of the 3D neuronal network over the long-term time span, we analyzed neuronal survival within the alginate scaffold (see Methods) and the physiological activity of the networks over a temporal window of approximately two months. As reported in Fig. 1e, in the hydrogel cultures we observed approximately 80% viable cells at 7 DIVs; this value slightly but not significantly decreased to approximately 72% at 56 DIVs. Moreover, we analyzed the development and stability of the network activity by fluorescence calcium imaging (in terms of the network’s mean firing rate), starting from 7–14 DIVs, when complete neuronal maturation (cell polarization, and membrane expression of receptors and ion channels) has occurred and the first network connections are established. To detect calcium fluctuations in the neurons during the first days of culture, we labeled some cultures with Fluo4-AM dye; at later DIVs (>10 DIVs), other cultures were infected with an adenoviral vector to induce stable long-lasting expression of the genetically encoded calcium sensor GCaMP6s. We did not infect the networks at early DIVs because obtaining sufficient expression of the GCaMP6s sensor even at 7 DIVs requires high viral vector concentrations^29^, and we aimed to minimize stress in the neuronal cultures during the first days of network development so we could reliably monitor the long-term maturation of the neuronal network activity at late stages of culturing.

The cell density in the 3D cultures was suitable for avoiding cell clustering, and it allowed the optical detection of single cell somata, thus making it possible to monitor the long-term calcium activity of the 3D neural networks by using a wide-field microscope (supplementary Video S2). The fluorescence signals of both the Fluo4-AM and the GCaMP6s sensors were mainly concentrated in the cell soma. Indeed, the signal intensity in the soma masks the signal coming from neuronal processes, thus allowing the specific detection of the fluorescent signals from the cell soma within the acquired images (see supplementary Video S2). Furthermore, the adopted image thresholding procedure discards thin and elongated fluorescent areas through dilation and erosion procedures (see Methods). In 3D cultures, a mean firing rate (MFR) was established and maintained over a time window of 63 DIVs (Fig. 1f). Moreover, 3D cultures showed spatial patterns of activity involving distinct cell clusters (see the highlighted regions of activity in Fig. 1g and the respective supplementary Video S2), thus indicating the development of complex and heterogeneous activity within the 3D networks.

### Tunable extended depth of field microscope: wide-volume functional imaging of 3D neuronal networks

**Figure 2:**
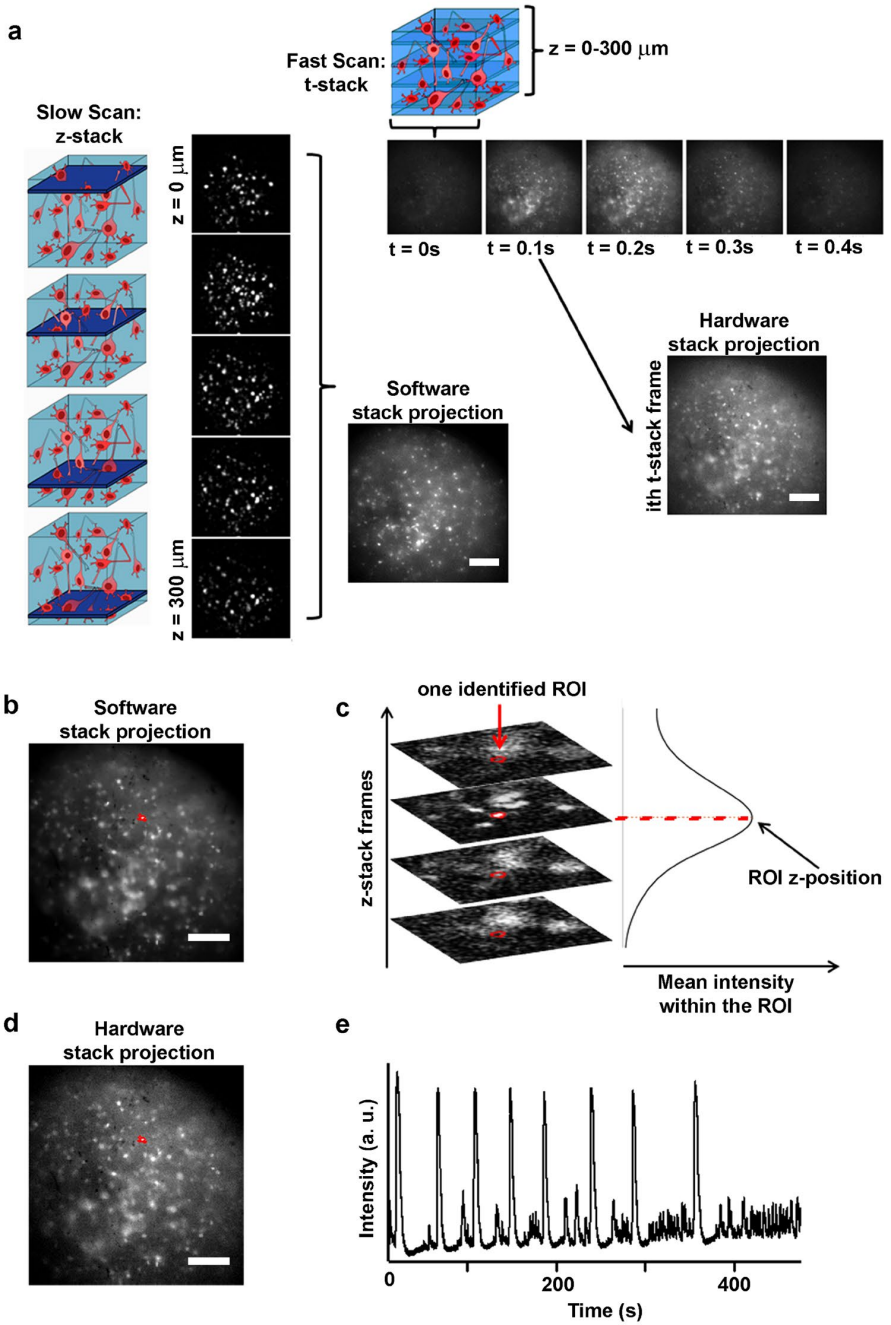
Scheme of the wide volume imaging protocol. (**a**) On the left, a cartoon illustrates the slow axial scanning imaging condition, and the corresponding images constituting the acquired z-stack. The z-stack is then projected on a single image by software, in order to produce an extended depth of field image. On the top, a cartoon illustrates the fast axial scanning imaging condition, and the corresponding images constituting the acquired t-stack. Each image (ith image) of the t-stack represents an extended depth of field image of the same sample portion scanned and acquired in the slow scan condition; (**b**) Cell somata are identified on the software projection of the z-stack as ROIs (a ROI is highlighted in red). The images were acquired on a cortical culture at 24 DIVs; (**c**) Identification of the z position of the highlighted ROI. The mean intensity within the ROI is computed in each frame of the z-stack. The computed mean intensity versus image frames reaches a maximum value when the cell is in focus; (**d**) The same ROIs detected in the software projection image are over imposed on the hardware aided projection of the cortical culture of panel b (the ROI is also highlighted in panel b); (**e**) The mean intensity of the ROI is computed in each frame of the t-stack, in order to obtain the plot of fluorescence intensity versus time, representing the cell detected calcium activity. Bars are 150 µm.

Although we were able to monitor the overall activity of the 3D networks using a wide-field microscope (on a large FOV of approximately 2.4 × 1.8 mm), we could not resolve the relevant third dimension of the networks. Therefore, to monitor and resolve the 3D activity of the neural networks at single-cell resolution, we developed a tunable extended depth of field (TEDOF) microscope. The optical layout of the system was a modified wide-field microscope with a phase-modulated detection pathway (supplementary Fig. S2). This phase modulation, achieved through a lens with tunable focal length, allowed inertia-free axial scanning of the sample that could also be tuned to an extended depth of field imaging. Once the field of view was chosen, it was possible to set the extreme positions of the axial scanning and to independently control the velocity of the axial scanning and the charge-coupled device (CCD) camera frame rate. This setup allowed us to operate in two configuration settings. In the first configuration, the CCD frame rate was higher than the axial scanning velocity. Using this configuration, we could acquire distinct images for several axial positions of the sample plane projected on the CCD and thereby obtain a z-stack series (Fig. 2a and supplementary Video S3). Analysis of the z-stack with a standard defocusing approach^38^ provided 3D localization of the sample features (Fig. 2b,c). In detail, through an automated analysis algorithm, we produced a single projection image of the z-stack; this was converted to a binary image by histogram thresholding. Then, the analysis algorithm identified several regions of interest (ROIs), representing the cell somata and their transverse positions in the projected image (Fig. 2b). The axial position of the ROIs was extracted by computing the mean fluorescence intensity within each ROI in all the frames of the z-stack. The axial position of the neurons was identified as the frame position at which the mean fluorescence intensity of the corresponding ROI reached its maximum value (Fig. 2c).

In the second configuration, the axial scanning velocity was much higher than the CCD frame rate, making it possible to collect, in one image frame, the fluorescence signals coming from all of the scanned sample planes (Fig. 2a). In other words, this experimental configuration produced an extension of the depth of field of the microscope (Fig. 2d); thus, we could collect a time-lapse series (t-stack) that allowed us to follow the calcium fluctuations of all the neurons within the scanned volume (supplementary Video S4). The fluorescence trace of each neuron could be extracted from the t-stack as the mean intensity value within the corresponding ROI in each time frame (Fig. 2e). It is worth noting that the neuron calcium traces shown in Fig. 2e include peaks with distinct maximum intensity values, indicating heterogeneous activity of single neurons within the 3D network.

### Efficient denoising of calcium traces and detection of calcium fluctuation events through a modified Perona-Malik filter

To analyze the acquired neuronal calcium traces, we designed a novel edge-enhancing denoising algorithm that efficiently detected calcium fluctuation events. Among the distinct types of filters, an important class of denoising techniques is based on the use of partial differential equations (PDEs) with an anisotropic diffusion coefficient, also referred as the Perona-Malik diffusion coefficient^39^. These types of filters, which are mainly adopted in image processing, allow the reduction of noise while preserving characteristic image features. Indeed, in these filters, the diffusion coefficient acts as an edge-seeking function that performs smoothing within slow varying regions and prevents smoothing across fast varying regions such as lines and edges. We propose to use this type of filter to remove the noise from the acquired calcium traces while preserving fast varying fluctuation events associated with the physiological activity of the neurons to allow their reliable automated detection. However, the anisotropic diffusion of the Perona-Malik filter, which is regularized by a so-called edge-stopping function related to the local gradient of the signal, is not suitable for every type of trace. Indeed, when the magnitude of the gradient of the trace is higher in the presence of noise than in the presence of significant edges, the Perona-Malik algorithm is not successful in removing the noise. Therefore, we propose a modified Perona-Malik filter with an edge-stopping function that depends on both the local and total magnitude of the gradient of the signal in a defined shifting window of the trace (see Methods). To validate the proposed novel Perona-Malik filter, we applied it to simulated fluorescence calcium traces and compared its performance with that of the classical version of the Perona-Malik filter.

**Figure 3:**
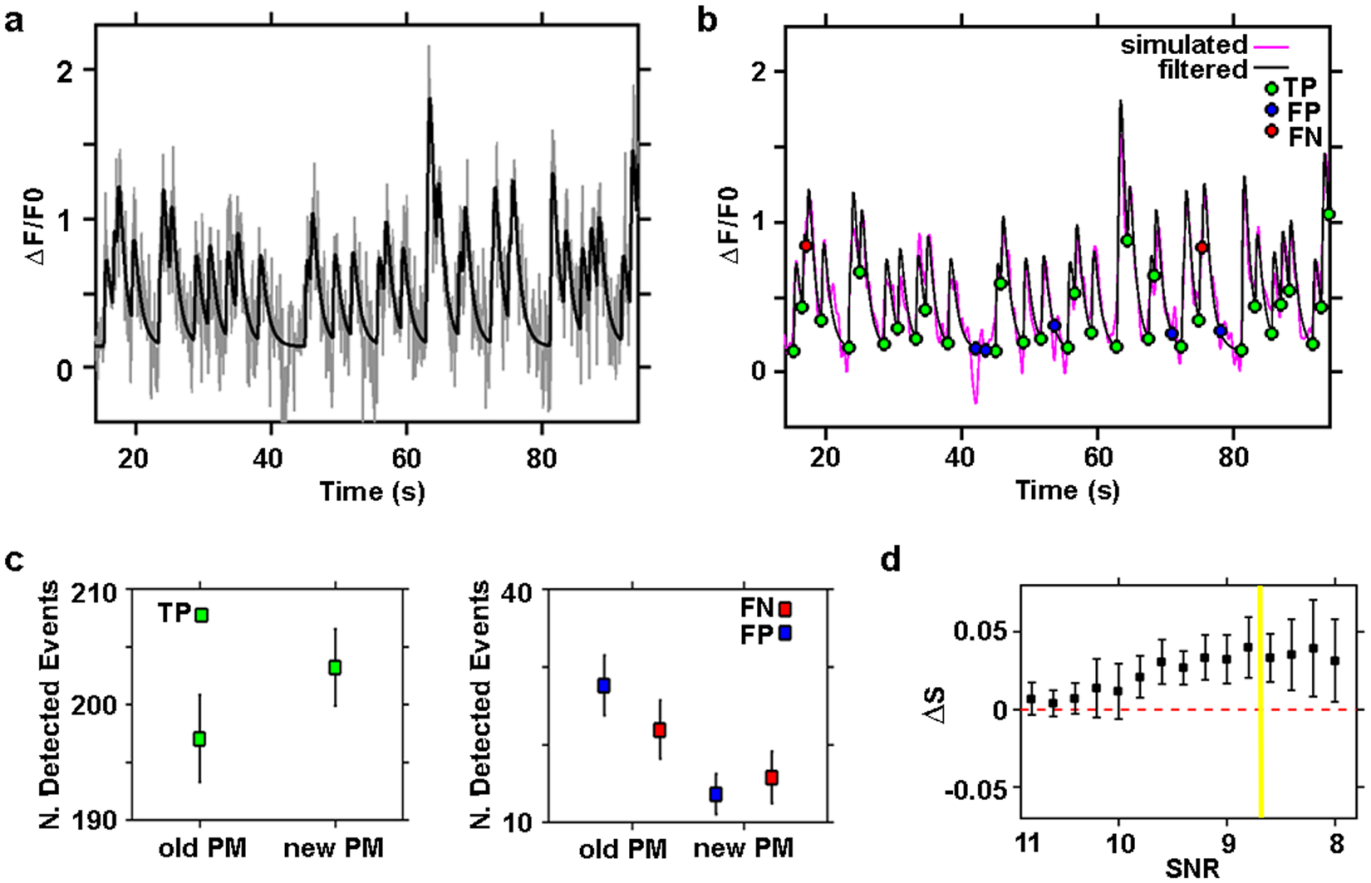
Detection efficiency of the novel Perona-Malik filter. (**a**) Simulated neuronal calcium trace (black line) with added noise (gray line), SNR = 9; (**b**) Simulated neuronal calcium trace (black line) shown in (**a**), with superimposed filtered trace (magenta line) obtained from the trace with added noise (gray line) shown in panel (a). The green, blue and red circles indicate the onset time of true (TP) and false (FP) detected events, and the onset time of non-detected events (FN) respectively, through the novel Perona-Malik filter; (**c**) quantification of the classical Perona-Malik filter (old PM) and novel Perona-Malik filter (new PM) performance in detecting true calcium events (TP) on left panel. On the right panel, comparison of the two filters in missing true events (FN) and detecting false events (FP). The quantification of the performance of the filters was done on 30 simulated traces with SNR = 9. Data are shown as mean ± std (n = 30 simulated traces); (**d**) Comparison of the two filter sensitivity, quantified as sensitivity difference (ΔS), with respect to decreasing levels of signal to noise ratio (SNR). The sensitivity S of the filters was computed as the ratio between the number of detected true events (TP) divided by the total number of events within the traces (TP + FN). Data are shown as mean ± std (n = 30 simulated traces for each SNR value).

In Fig. 3a, we report an example of simulated fluorescence calcium fluctuations within a single neuron (in black) with additive white noise (gray trace). We set the noise variance to obtain a signal-to-noise ratio (SNR) similar to that of the experimental data (see Methods). In Fig. 3b, we show that the filtered signal (in magenta), obtained from the signal with noise (gray trace in Fig. 3a) well preserves the fluctuation events of the original simulated trace (in black). The green, blue and red circles in Fig. 3b indicate, respectively, the onset times of true and false detected events and the onset times of real events that are not detected by the new filter. To verify that the new Perona-Malik filter improves the detection of calcium events of the the classical Perona-Malik filter, we compared the performances of the two filters on simulated calcium traces (see Methods). We quantified the number of true and false positive (TP and FP, true and false detected events, respectively) and the false negative (FN, missed events) events detected by the classical and the novel Perona-Malik filters (indicated as oldPM and newPM, respectively). The results reported in Fig. 3c confirm the better performance of the modified Perona-Malik filter in terms of both the number of detected TP (newPM detects more TP) and FN (newPM produces less FN) and highlights its comparable results in terms of FP. Furthermore, we compared the sensitivity of the filters’ performance with respect to the SNR of the traces. In Fig. 3d, we plot the difference (ΔS) of the sensitivity (S) of the two filters computed as the total number of detected real events (TP) divided by the number of real events within the traces (TP + FN) as a function of decreasing value of the SNR. The results show that ΔS (equal to S_newPM_ minus S_oldPM_) is > 0 for the reported SNR range; thus, the plot indicates that the modified filter outperforms the classical one.

Considering that from our traces we estimated a SNR of approximately 8.7 ± 0.6 (mean ± standard deviation estimated over n = 200 distinct traces), we thus validate the use of the novel filter to analyze our data. We applied it to the acquired single cell calcium traces to characterize and quantify the activity of the 3D networks. The raw traces of the neurons extracted from the t-stack were baseline-corrected and normalized to calculate the normalized fluorescent calcium signals, which are indicated as ΔF/F0 (F: fluorescence intensity in a.u.). The baseline F0 of the traces was automatically estimated using a linear diffusion filter that evaluates only the slow varying component of the trace by setting a large time window (time window length = 30 s). The normalized traces were then smoothed using the modified Perona-Malik filter. We note that due to the form of our filter and the linearity of the derivatives, the smoothness process does not alter the normalization of the calcium signal.

**Figure 4:**
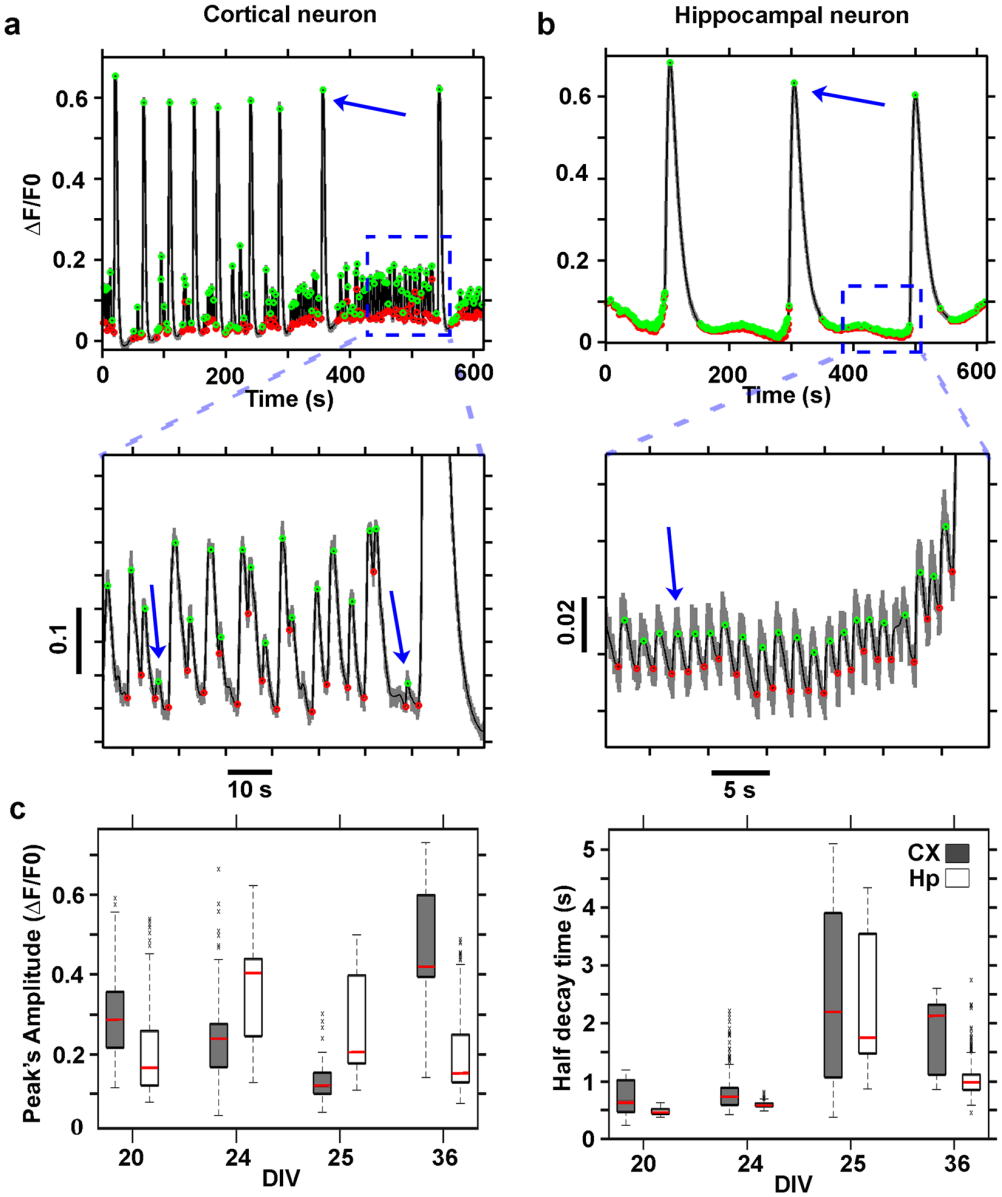
Calcium trace denoising and event detection. ΔF/F0 trace of a cortical neuron of a cortical 3D network at 24 DIVs (**a**) and of a hippocampal neuron of a hippocampal 3D network at 36 DIVs (**b**). The denoised traces are shown in black while the onset and offset of the events are marked, respectively, in green and red. The insets, below the graphs, show the small amplitude events which were detected; In (**c**) Box plots illustrating the distribution, at distinct DIVs, of peak amplitudes (left panel) and the half decay times (right panel) of the detected fluorescence fluctuation events of cortical (CX) and hippocampal neurons (Hp) traces. The value distributions in the box plots have been computed on all the detected traces (691 ÷ 1495 distinct cell traces) of n = 6, 7, 8, 4 cortical and n = 4, 3, 6, 6 hippocampal distinct hydrogels at respectively 20, 24, 25, 36 DIVs.

In Fig. 4a,b, we report two representative traces of a cortical and a hippocampal neuron, respectively (see also supplementary Fig. S5 showing two representative raster plots of the activity of cortical and hippocampal 3D networks). It can be noted that both the raw traces (shown in gray) present high-intensity peaks interspersed with small fluctuation events. Traces from cortical neurons had small events with variegated amplitude levels. In contrast, hippocampal neurons featured trains of stereotyped small calcium fluctuations preceding the high- amplitude events (enlarged insets in Fig. 4a,b). The modified Perona-Malik filter accomplished efficient trace smoothing (superimposed black traces shown in detail in the insets of Fig. 4a,b) while preserving both small and big fluctuation events with their representative peaks and allowed the reliable detection of the onset and offset times of the events (green and red circles, respectively, in Fig. 4a,b). Indeed, it is worth noting that both in the cortical and hippocampal neuron traces the detection algorithm reliably detects big events with peak intensities of approximately 0.6 ΔF/F0 and small events with intensities of approximately 0.05 (inset of the cortical neuron trace) and 0.02 (inset of the hippocampal neuronal trace) ΔF/F0, thus providing efficient detection of events approximately 12 (indicated by blue arrows in the inset in Fig. 4a) and 30 (indicated by the blue arrow in the inset in Fig. 4b) times smaller than the largest events present within the same traces (indicated by the blue arrows in Fig. 4a,b, respectively). Among the different types of genetically encoded sensors, we used GCaMP6s because it has been reported to provide a high signal-to-noise ratio for the detection of fluorescence fluctuation events whether these events are associated with one or ten action potentials (APs), thus yielding the highest fraction of isolated spikes detected at a false positive rate of 1%. Moreover, the use of GCaMP6s allows expression of the sensor over a period of 1–2 months without altering the functions of neuronal circuits^24^.

After detecting the events, we characterized each calcium transient in terms of its peak amplitude and its half- decay time; thus, we reconstructed the distribution of peak amplitudes and decay time values (Fig. 4c). The distribution of values reported in Fig. 4c (right panel) agrees well with the previously published calibration values for the indicator GCaMP6s, thereby correlating the half-decay time of the detected fluorescence peaks to the number of underlying neuronal action potentials: approximately 500 ms for a single action potential (AP) and 1.8 s for 10 APs^24^.

Single traces from cortical and hippocampal neurons were both characterized by low- and high-intensity peak events with short and long half-decay times, respectively. As already reported in the literature for another model of 3D cortical cultures^29^, high peak fluctuation events with long half-decay times (overcoming the typical GCaMP6s time response to a single AP of approximately 500 milliseconds) are associated with bursting or super-bursting activity of the neurons. The decay time constant was systematically higher (p < 10^−6^) in the cortical cell cultures than in the hippocampal cultures (Fig. 4c), and the decay time constant peaked at 25 DIVs for both preparations. Moreover, a previous study showed that network activity in standard 2D cell culture recordings tends to be stronger and more coordinated in hippocampal than in cortical networks^40, 41^. The latter result is consistent with our findings and suggests that the longer decay time constant observed for the cortical data might correspond to less synchronized activity with respect to the hippocampal networks.

The amplitude of the response (Fig. 4c, left panel) displayed a less systematic change across DIVs. In particular, the amplitude was higher for the hippocampal network at intermediate DIVs, suggesting that the activity in these networks is more coordinated than that in cortical networks during these phases.

Although the amplitude and decay time constant provide some information on the emergent activity of the network, a more thorough analysis is required to characterize the state of the network. A detailed analysis will be presented in the next section.

### Characterization of the 3D neuronal network activity

**Figure 5:**
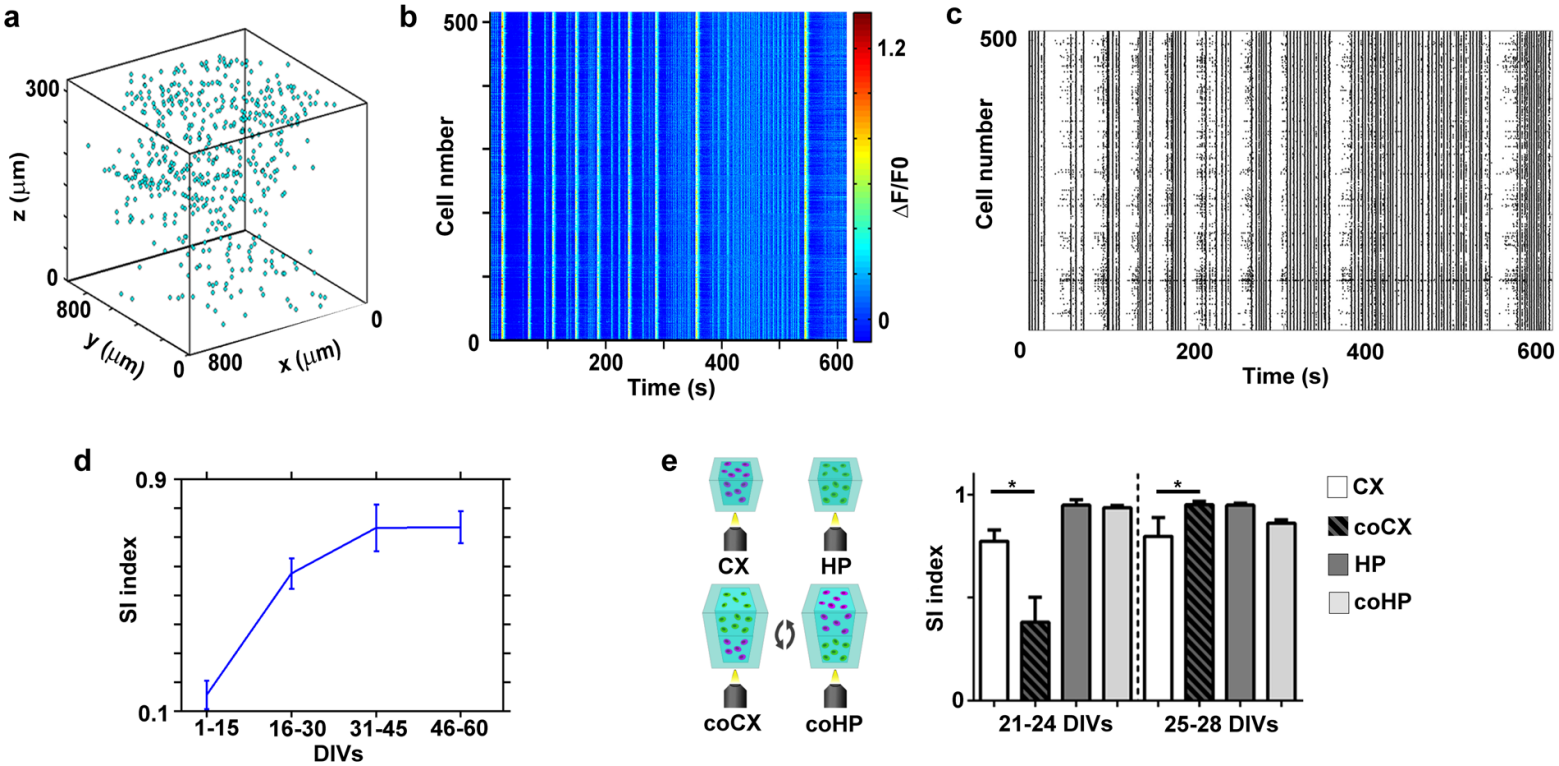
Structural and functional reconstruction of a representative 3D cortical network. (**a**) 3D representation of the cell somata positions, within the acquired volume portion of the 3D cortical neuronal network at 24 DIVs; (**b**) ΔF/F pseudo-color plot of the identified cells of the network shown in **a**). (**c**) raster plot of the same network as in a) and b) constructed after the automated calcium event detection analysis. (**d**) Plot showing the synchronicity index (SI) of 3D cortical cultures over time; data shown are the mean values ± standard errors (n = 17 for each group of DIVs); (**e**) On the left, a scheme of the optical monitoring layout of the compared conditions is shown (CX = cortical network analyzed in single cultures, HP = hippocampal network analyzed in single cultures, coCX = cortical network analyzed in layered co-cultures, coHP = hippocampal network analyzed in layered co-cultures). On the right, bar plot showing the synchronicity index (SI) of single and layered co-cultures at early and late DIVs; data shown are the mean values + standard errors (n = 7 for each group at 21–24 DIVs, and n = 13 for each group at 25–28 DIVs). Note that the data were grouped differently than in Fig. 4 to facilitate the comparison among different preparations (e.g. we managed to record only one co-culture at 36 DIVs).

After extracting the events associated with the single neuronal calcium traces, we analyzed the overall network activity. Given the optical transparency of the 3D hydrogel, we could reproducibly scan 300 µm depth of the sample with a good signal-to-noise ratio. Through slow axial scanning of the sample, we extracted the 3D architecture of the neuronal network (Fig. 5a), and through fast axial scanning, we monitored the activity of approximately 300–500 neurons in a volume of 800 × 800 × 300 µm (Fig. 5b). Thereafter, using the novel detection algorithm, we reconstructed the network activity (Fig. 5c). In supplementary Fig. S5, we report two representative raster plots of cortical and hippocampal neuronal networks; in another example, we note that the raster plot of cortical networks presented complex temporal patterns with mixed asynchronous (appearing as sparse clusters of black dots) and synchronous (appearing as straight vertical lines) activity and with distinct frequencies of bursting activity. Otherwise, the hippocampal cultures were characterized by a gradual development of bursting activity interspersed with silent periods of the network and by an overall time-preserved bursting pattern. To verify that the detected raster plots were representative of functional network activity, we performed control experiments on cultures treated with antagonists of synaptic receptors and sodium channels (supplementary Fig. S6).

To further investigate the development of activity in the 3D cultures, we analyzed the time progression of synchronous activity levels within the cortical networks and quantified it in terms of a synchronicity index (SI) (see the Methods section). We observed a gradual long-term shift from asynchronous (till 15 DIVs, SI = 0.15 ± 0.05) to partially synchronous (16–30 DIVs, SI = 0.57 ± 0.05) and highly synchronous activity (from 31 DIVs, SI = 0.73 ± 0.05), with an overall increasing trend of the SI (Fig. 5d). Furthermore, we compared the SI of cortical and hippocampal neuronal networks of single or layered co-cultures to assess how their network dynamics could be affected by the layered co-culture structure. Although we could simply flip the agar mold horizontally, as shown in Fig. 1b, to obtain optical access to both the cortical and hippocampal layers, it was not possible to easily identify the culture interface without using additional fluorescent markers to discriminate the two distinct cell types, both of which expressed the green fluorescent GCaMP6s sensor. Therefore, we decided to vertically flip the agar mold (see the cartoon in Fig. 5e) to monitor the cortical and hippocampal layers. Considering that the optical penetration depth of the TEDOF system was approximately 300 µm and that each network layer was approximately 1 mm thick in the axial direction, in the experimental configuration approximately 700 µm of neural tissue separated the inspected portion of the network from its interface with the other distinct neuronal culture type. In contrast, the FOV of the TEDOF microscope was approximately 800 × 800 µm in the transverse direction, which is approximately the size of a layer when the mold is flipped horizontally. Therefore, in the adopted configuration, we were careful not to mix the activities of the distinct layers. Moreover, we did not face problems related to fluorescence bleed through channels and the consequent increase in the fluorescence background, which occurs when using multiple fluorescent proteins labelling^42^ and could deteriorate the monitoring and detection of neuronal network activity.

We observed that hippocampal networks presented a higher SI than cortical ones (bar plot in Fig. 5e) and did not show significant differences between single and co-cultures (21–24 DIVs, p = 0.383, 25–28 DIVs, p = 0.479). However, cortical networks showed remarkably different SIs in the two configurations. In particular, cortical SI was significantly lower in co-cultures than in single cultures at early time points (21–24 DIVs, p = 0.015), whereas it became significantly higher and comparable to that of the hippocampal networks in the co-culture condition at later time points (25–28 DIVs, p = 0.0015).

**Figure 6:**
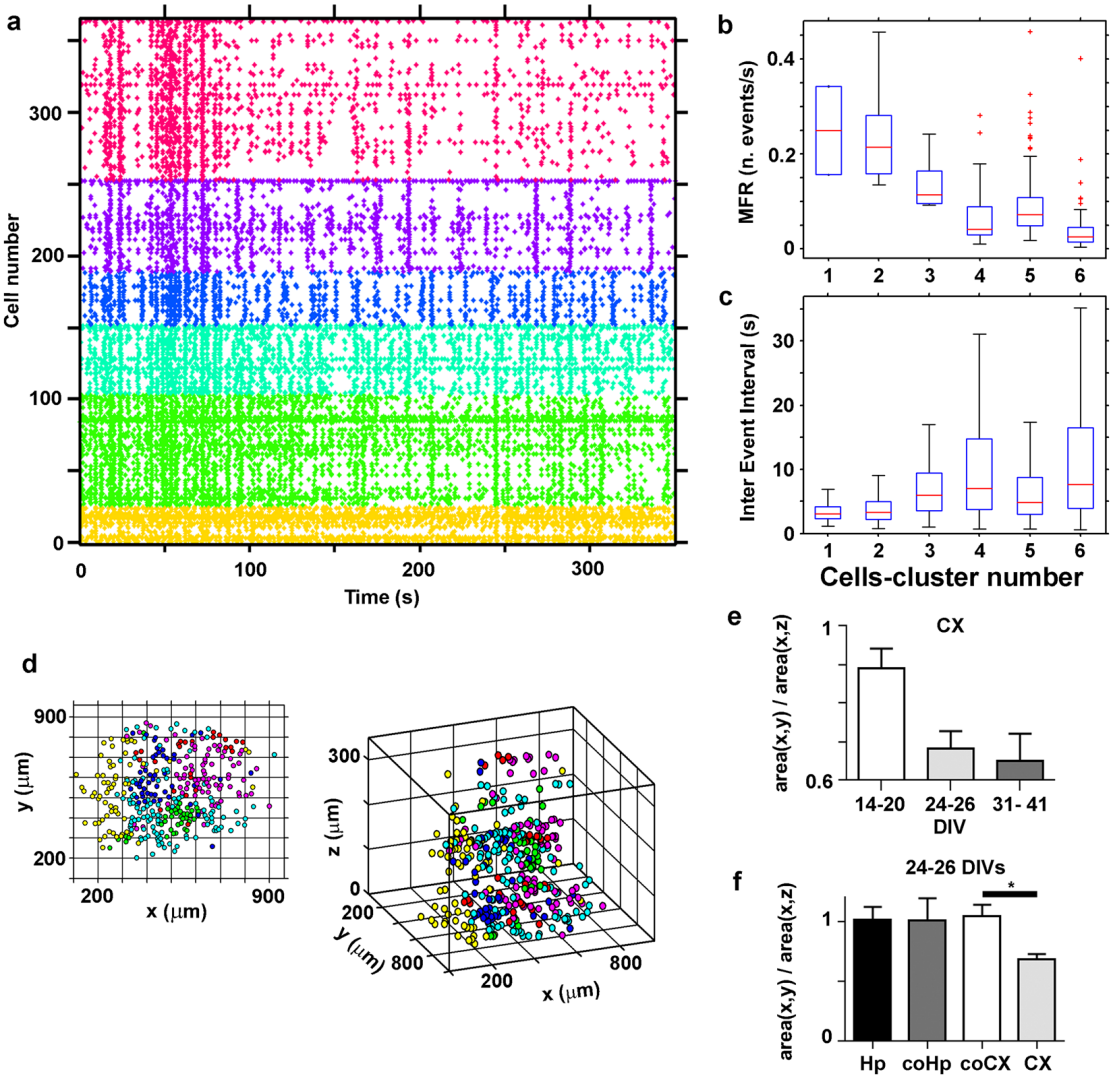
Clustering of spike pattern activities. (**a**) Raster plot of a 3D cortical network at 26 DIVs, with cells ranked according to the functional clustering. Cells are divided into 6 classes according to their activity profiles over time; (**b**) Comparison of the distribution of the Mean Firing Rate (MFR) among the 6 detected clusters of the network of panel a); (**c**) Comparison of the distribution of the Inter Events Intervals (IEIs) among the 6 detected clusters of the network of panel a). Boxplots in (**b** and **c**) show the median and the interquartile range. Outliers are considered all those values whose distance from the first (or third) quartile is larger than 1.5 times the interquartile range. The results in (**b** and **c**) show that the calcium activity is significantly different between each pair of the identified clusters (Mann-Whitney U-test, p < 0.05, two-tailed); (**d**) Spatial distribution of the cell somata colored according to the clustering (x-y view on the left, x-y-z view on the right) of the network of panel a. The x-y view suggests that clusters are spatially localized. The 3D view, instead, suggests that they are well distributed along the third dimension; (**e**) Quantification of the ratio of average area covered by each cluster (divided by the number of cells belonging to the cluster) in the transversal view and in the coronal-sagittal view of 3D cortical cultures (indicated as CX in the figure panel). The covered area differs significantly between the two views at DIVs > 14 (Mann-Whitney U-test, n = 13, 14, 9 total number of clusters identified in three distinct experiments of the groups of 14–20 DIVs, 24–26 DIVs and 31–41 DIVs respectively, p = 0.7869, p = 0.0085 and p = 0.0117 respectively, two-tailed) for cortical neuronal networks in panel e). Data are shown as mean + standard errors. (**f**) The same quantification reported in panel e), for single hippocampal (Hp) cortical (CX) culture, and cortical (coCX) or hippocampal networks (coHp) analyzed in layered co-cultures at 24–26 DIVs. (Mann-Whitney U-test, n = 13, 8, 9, 14 total number of clusters identified in three distinct experiments of the groups Hp, coHp, coCX and CX respectively, p = 0.0317, two-tailed).

To validate the existence of distinct spatial patterns of activity in 3D networks (see the example reported in Fig. 1g), we further applied a community detection algorithm (see Methods section) to the data in the raster plots. In Fig. 6a, we report an example of detected clusters (indicated by different colors in the raster plot) within a cortical neuronal network at 26 DIVs. In this way, we could reliably detect varying numbers of groups (min. 4 – max. 6 in n = 3 distinct experiments for each condition reported in Fig. 6e,f) representing neuronal subpopulations with similar temporal patterns of activity within the network. The neuronal clusters detected within the network presented different MFR and inter-event interval (IEI) distributions, as reported in the box plots in Fig. 6b,c. We confirmed that each cluster represented a group of neurons with significantly different features through multiple comparisons of the corresponding mean values of the MFR and IEI. After recognizing the neuronal clusters through their temporal patterns of activity, we retrospectively reconstructed the spatial arrangement of the neuronal subpopulations within the network (Fig. 6d). We found that cells belonging to the same community were spatially localized in the transverse plane, whereas they were well distributed along the vertical dimension of the sample (Fig. 6d). Indeed, the area in which the cells clustered was significantly smaller in the transverse plane than in the sagittal-coronal plane (Fig. 6e), indicating a columnar organization of the cell clusters, which was just perceptible (ratio of transverse versus sagittal-coronal area equal to ~0.9, p = 0.787) at 14–20 DIVs. The clusters reached a stable and significant elongated organization at approximately 24–26 DIVs (ratio of the cluster areas equal to ~0.7, p = 0.008), and this was preserved at later DIVs (30–40 DIVs, p = 0.012). However, although the clusters were located within a limited portion of the transverse plane, they were not spatially segregated (i.e., the silhouette coefficient was lower than the 95^th^ percentile of the silhouette coefficients of the randomized labeled transversal cell data points). The latter indicates that the clusters were intermixed in almost all preparations.

The absence of a columnar organization of the neuronal clusters at early stages indicates *per se* that the obtained result is not an artifact of the imaging approach. However, we further verified this by applying a demixing algorithm (previously reported by Paninski *et al*.^26^) that can be used to discern the activity patterns of neuronal cells that overlap in the axial direction when parallel monitoring of several optical sections is performed^21^. If the columnar organization is due to the overlapping of cells, it should not be obtained after the demixing step. The ratio of the transverse-axial area of the clusters measured in the example reported in Fig. 6a–d decreased after the demixing procedure (before demixing, it was equal to 0.75 ± 0.31, and it decreased to 0.55 ± 0.28 after the application of the demixing algorithm). Indeed, the demixing step could ameliorate the trace detection of the ROIs corresponding to distinct cells aligned along the optical axis and thus improve the estimate of the columnar organization; in any case, the result confirms that the detected neuronal organization is not due to imaging artifacts.

We performed a similar analysis of hippocampal networks of single cultures and of hippocampal and cortical networks of layered co-cultures at 24–26 DIVs (the time window at which the columnar organization emerges; see Fig. 6e). The results in Fig. 6f indicate that hippocampal networks (in both single and co-culture condition) did not present the columnar organization of the single cortical cultures and that cortical networks lost the columnar organization when they were at the interface with a hippocampal culture.

To investigate how neuronal activity is transmitted in the 3D networks, we performed an additional analysis. We used the transfer entropy (TE), which, unlike the similarity measure (Fig. 6), can be used to investigate the direction of information flow in a network.

**Figure 7:**
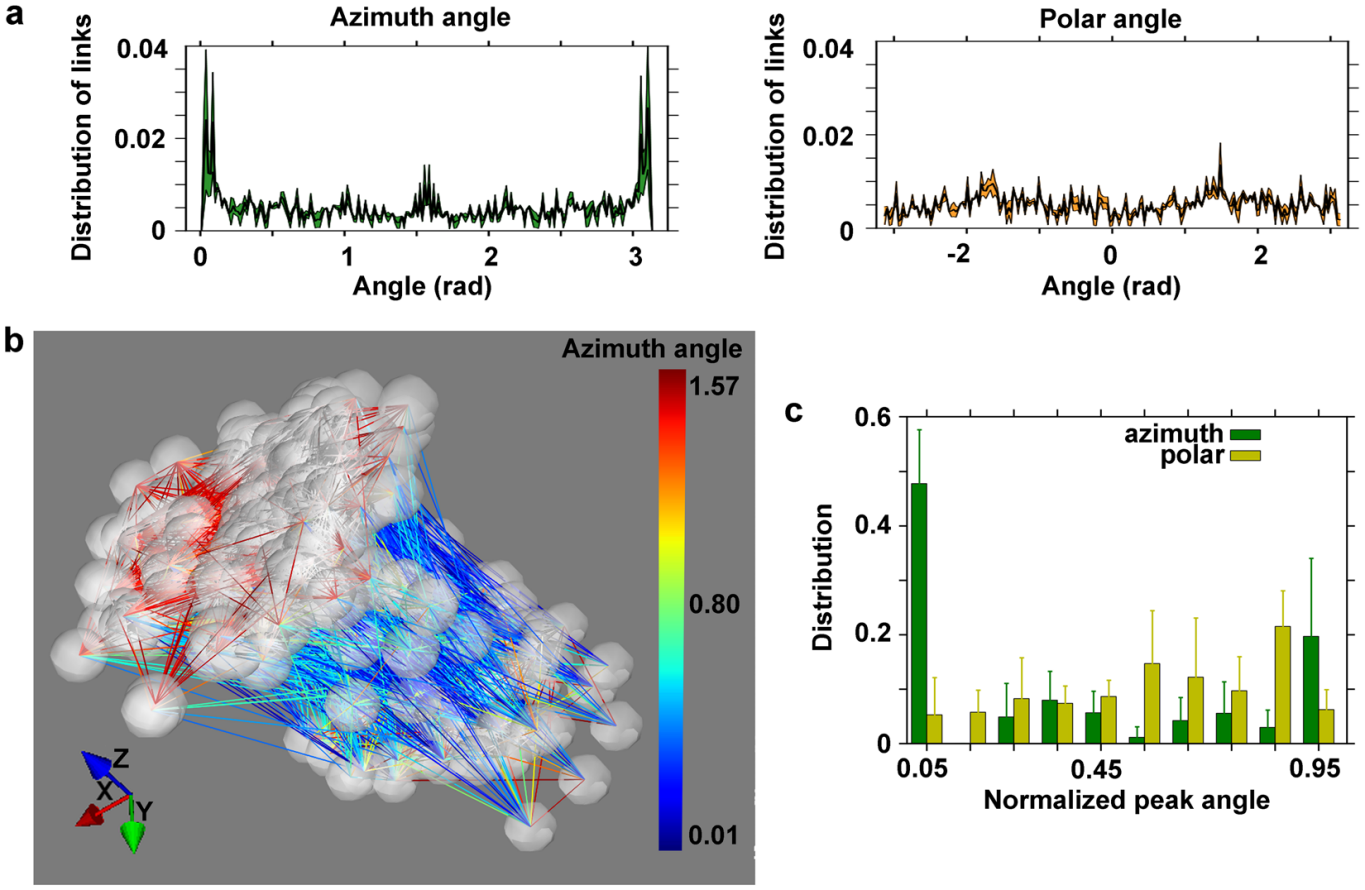
Properties of the functional graph. (**a**) The azimuth (left panel) angle of the detected functional links of a cortical neuronal network (32 DIVs) has a peak at 0, π (i.e. along the z-axis, the vertical axis) and at π/2 radian. The corresponding polar (right panel) angle fluctuates around the value 0.005. (**b**) Illustrative plot of the 3D cell culture at 32 DIVs of panel a), showing the predominance of vertical links (blue) with the coexistence of horizontal links (red). The vertical links (blue) correspond to the peaks of the azimuth angle at 0 and π radians. The horizontal links (red) correspond to the peak at π/2 radians. Note that the dimensions of the plot are not to scale and have been stretched to evidence the orientation of the links in the network, and all the recorded cells of the network on the entire volume view of the optical system (800 × 800 × 300 µm) are reported. (**c**) The vertical orientation is a general feature of the studied 3D cortical single-cultures. Population summary histogram of the distribution of normalized polar and azimuth orientation angles of the detected neuronal links. Data shown are the mean values + standard errors (the groups consisted of 20, 22 24 and 13 cell cultures, respectively).

We first identified significant functional connections among neurons on the same transverse plane as well as across different z-planes, supporting the existence of a real 3D functional structure (see the example reported in Fig. 7b). Although this analysis also showed that the intra-cluster mean connectivity strength was significantly higher than the inter-cluster connectivity strength (1.30 ± 0.04% higher intra-connectivity strength, p = 0.016, one-sided test), it indicates the existence of both columnar and trans-columnar communication within 3D cortical cultures^43^.

Inspired by the results regarding the functional columnar organization of the cultures (see Fig. 6), we quantified the azimuth and polar angle distributions of the orientations of the identified functional links. In Fig. 7a, we report the distribution of the azimuth and polar orientation angles (left and right panels, respectively) of the detected functional links within a single cortical culture at 32 DIVs. We found that they were preferentially oriented along the z-axis (i.e., in the vertical direction, with peaks at an azimuth angle of 0 and 3.14 radiant). Figure 7b presents an illustrative plot of a 3D cortical culture showing the predominance of vertical links (indicated by blue lines) together with the coexistence of horizontal links (indicated by red lines). Finally, to verify that the vertical orientation of the links is a general feature of the studied 3D cortical single cultures (DIVs 24–41, n = 6), we performed a population analysis. Figure 7c shows a population summary histogram of the distribution of the normalized polar and azimuth orientation angles of all the detected neuronal links within the 3D cortical single cultures grouped into four consecutive time intervals: 7 to 13 DIVs, 14 to 20 DIVs, 21 to 28 DIVs and 29 to 46 DIVs. The peak in the distribution of the azimuth angle indicates a preferential orientation of the links along the vertical axis, whereas the polar distribution of the angles does not indicate a preferential orientation of the links in the transverse plane of the neuronal networks. The population analysis confirmed that the vertical orientation of the links is a common feature that is observed throughout the identified range of DIVs (24–41 DIVs) of single cortical cultures (see Fig. 6f).

## Discussion

Currently, a multidisciplinary approach that includes experimental and theoretical modeling, computational neuroscience, and engineering is essential to shed light on the relation between neural activity and behavior and to identify the origin of brain dysfunction in disease. Currently, only *in vitro* systems allow correlative methods to be applied in high-throughput screening assays. Consistent with this, great advances have been made in biomimetic approaches to the production of organ architectures on chips^44^. Although several organ-on-chip models have been designed, most of them rely on 2D interconnected compartmentalized structures^45^; these models allow studying the long-range interplay between compartments that is mediated by soluble factors but fail to resemble real tissue-tissue interfaces. Among them, only a few brain-on-chip models have been proposed, and none of these have the appropriate properties to fully mimic the *in vivo* extracellular matrix.

However, it is well recognized that 3D *in vitro* cultures represent a further step in bridging the gap between *in vivo* and *in vitro* studies, and several approaches based on controlled cellular assembly or self-assembly are currently being developed^46^. Although cellular self-assembly approaches^47^ may provide more realistic mimicking of *in vivo* scenarios, they fail to control cell distribution for engineering and modeling specific brain regions or to control cell density for gaining favorable optical properties. Indeed, functional imaging of neurosphere cultures presents the same challenges as those presented by native tissues, and it is usually restricted to single optical sections.

Here, we propose an *in vitro* system of 3D neuronal cultures embedded in a hydrogel scaffold. This culture system, which has good optical properties and an elasticity^17^ resembling that of brain tissue^48^, has been extended to produce controllable layered structures. The hydrogel scaffold supported neuronal maturation, as indicated by the colocalization at late DIVs of vGLUT1 and vGAT transporters on the same nerve terminals^49, 50^, and had a density per single neuron of excitatory and inhibitory vesicular transporters comparable to that encountered *in vivo* ^36^. Moreover, the embedded neuronal networks exhibit well-tolerated long-term expression (two months) of the GCaMP6s sensor. To the best of our knowledge, only in *in vivo* models the long-term expression of genetically encoded sensors has been reported for investigating network dynamics in time windows ranging from a few months to a year. This, together with the low cellular density of the culture, allowed monitoring of the 3D network activity of the cultures at high SNR with a high temporal bandwidth and down to single-cell resolution. Therefore, the experimental model of 3D networks described herein could represent an important advancement in tissue engineering^51^ and neuroscience research.

In our 3D cultures, we were able to monitor the activity of neurons at 300 µm depth with single-photon excitation and a wide-field detection configuration. Moreover, our simple TEDOF microscope provides continuous monitoring across the full scanned volume of the neural network rather than single points or focal planes, thus achieving ultra-fast wide volumetric imaging whose temporal resolution is limited only by the sensitivity of the detector. In addition, the ability to control the speed and extent of the sample axial scanning allows the monitored volume portion to be tuned to the characteristics of the sample and the sensitivity of the detector and to achieve cell soma 3D spatial localization and 3D fast monitoring of network activity within the same workstation. Indeed, although several distinct approaches, such as clearing tissue methods^52^, genetic multicolor labelling^53^, and correlative approaches^54^, are being developed to link the physiological activity of neuronal populations with their resolved connectome, they gain a posteriori information after a specific treatment of the sample, and/or they are performed on specific systems to obtain functional or structural information. Conventional scanning systems such as confocal and two-photon microscopes allow the acquisition of concomitant structural and functional information, but they provide limited temporal bandwidth for monitoring the activity of the network (resonant scanning systems achieve approximately 30 Hz temporal bandwidth). Novel optical architectures based on AODs, temporal focusing, and SLM are used to perform ultra-fast random access scanning or parallel detection of fluorescent signals, but they are complex, high-cost systems and demand very skilled users.

Although other optical configurations that extend the depth of field of the optical microscope have been successfully proposed, those configurations rely on the use of a fixed-phase mask in the detection pathway, which introduces image distortions requiring image deconvolution processing^27^. Moreover, such configurations have a fixed extension of the depth of field (~1 mm), which in some cases exceeds the dimensions of the sample itself and thus provides less efficient detection of the emitted fluorescence signals with a lower signal-to-noise ratio. Further, the fixed-phase mask does not allow switching back to conventional imaging. Other optical layouts affect both the excitation and detection pathways^28^, thus introducing a varying magnification factor of the microscope at distinct depths of the sample, which then requires image registration processing. The alternative use of fast motorized elements^29^ provides a temporal bandwidth of a few tens of Hz, which limits the number of planes (usually to two or a few planes) and the temporal resolution achieved by simultaneous monitoring of distinct optical sections. Moreover, motorized elements introduce problems regarding the mechanical stability and complexity of the whole optical system, and this becomes a relevant issue in developing automatized long-term monitoring devices. Therefore, the use of a tunable lens that allows tunable inertia-free axial scanning without image distortion and with a temporal bandwidth of up to 1 kHz represents a cheap and effective solution to performing multiplane imaging of 3D neuronal networks. Moreover, the proposed optical layout allows a straightforward switch from conventional to extended depth-of-field imaging. Considering that at least one back-and-forth fast scanning of the sample is sufficient to efficiently collect the fluorescence signals, the temporal resolution limit of the TEDOF is equal to the tunable lens bandwidth (approximately 1 kHz). In the present work, we achieved an exceptional wide-volume (0.19 mm^3^ volume) scanning rate of 65 Hz, which was dictated by the sensitivity of the CCD camera. The use of an sCMOS camera or more sensitive imaging devices could make it possible to reach the temporal limit dictated by the TL used here. Other tunable lenses, such as TAG lenses, provide a temporal bandwidth of several kHz, which could favor further improvement of the temporal resolution. However, such lenses do not always allow the speed of scanning to be controlled for performing both slow- and fast-scanning imaging and thereby combining 3D spatial and temporal information with the same system. Moreover, it has been shown that the GcAMP6s sensor provides an accuracy of approximately 8 ms for detecting the onset of a neuronal AP^42^; thus, a temporal resolution of a few hundred Hz is actually sufficient.

The major limits of the proposed TEDOF microscope depend on the illumination scheme, which is based on a single-photon wide-field layout. Although this simple and cost-effective solution allows conventional wide-field microscopes to be improved to gain 3D spatial and temporal information about the neuronal networks, it requires a low density of neuronal cells within the scaffolds to avoid cell clustering and massive overlapping of cells soma along the optical axis. In this regard, the application of powerful demixing algorithms^26^ could provide an effective solution. Another limitation related to the wide-field detection scheme is the discrimination of the neuropil from the soma of the cell. This is not always straightforward as the intrinsic depth of field of the objective used is approximately 10 µm; therefore, after image deconvolution processing^55^, it is not possible to resolve the subcellular compartments of the cells in the axial direction. In any case, high-resolution confocal systems based on temporal focusing approaches^33^ present an axial resolution of 13 µm, and the discrimination of the neuropil still represents a delicate task^26^. Nevertheless, it is worth noting that the proposed approach could be simply implemented as an add-on optical detection path with other illumination configurations such as two-photon structured illumination^56^, which is less prone to light scattering, suitable for *in vivo* imaging, and provides confocal subcellular resolution in the optical axial dimension^27^. For example, a two-photon structured illumination scheme based on a spatial light modulator could be exploited to set distinct focal spots on the soma of distinct neuronal cells expressing a genetically encoded calcium sensor within a 3D volume of the sample, and use of the independent TEDOF detection scheme could provide simultaneous monitoring of the fluorescence intensity fluctuations of the targeted cells over an axial extent tuned to match the axial extension of the excitation spatial pattern.

In the present work, we further present a novel denoising algorithm that allows efficient detection of both small and big calcium events and an analysis algorithm; together, these make it possible to conduct long-term characterization of the activity of *in vitro* 3D neural networks. Interestingly, the analysis of the spike pattern similarity (i.e., clustering) and the functional connectivity (i.e., TE), showed that 3D cortical neurons self-organize into columnar assemblies similar to those found in the neocortices of both invertebrates and vertebrates^57, 58^. In the brain, cortical columns emerge from the development and refinement of synaptic contacts that are formed following inputs from other brain regions (e.g., the thalamus)^59^. The role of this organization is still debated in the neuroscience field, and some authors feel that it could be a simple by-product of synaptic development^60^. Notwithstanding the differences with respect to the real brain, our cortical 3D cell cultures might organize in columns because synaptic specialization might still be sufficient to connect the network in columns; this represents an intriguing model for future verification.

One could argue that the axons grow following some optimization criteria of information flow that might underlie the development of layered brain regions (e.g., the cortex). The observed non-homogeneous connectivity could emerge from guiding forces (e.g., a chemoattractant gradient^61^) that are stronger in the vertical direction due to gravitation. Indeed, the 3D scaffold allows the formation of chemical gradients within the network, as soluble and slowly diffusing molecules could be locally trapped within the hydrogel polymer network. The integration of microfluidic systems^62^ could permit the study of the effects of controlled local release of chemical compounds in a 3D geometry. In contrast, such gradients could not be easily established and maintained in the bath of 2D neuronal cultures within Petri dishes. Another hypothesis is that during the gelation phase of the hydrogel gravitation could generate a concentration gradient of alginate and calcium ions that would translate into a vertical gradient of matrix stiffness that guides axonal growth and pathfinding^11^.

The fact that the columnar organization of neuronal subpopulations is not present in single cultures of 3D hippocampal networks or in cortical and hippocampal networks of layered co-cultures indicates that the columnar organization emerges from environmental cues to which only cortical cultures are sensitive and that the distinct hierarchical organization of developing hippocampal networks also influences nearby cortical networks. These observations led us to discard the hypothesis of a pure gravitational effect and thus also to consider the role of network activity in the generation of hierarchical network organization. In this regard, we monitored the physiological activity of the distinct layers; the results indicated that single hippocampal networks display more synchronous activity than cortical networks. It also appeared that the more synchronous activity of hippocampal networks influenced the development of intrinsic activity in the cortical networks in the co-culture configuration. To explain these results, we could hypothesize a master-slave arrangement^63^ of the co-cultures wherein the intrinsically more synchronous hippocampal layer (the master) seeds entropy in the early stages of the less synchronous cortical layer and later drives the latter toward highly synchronous activity. To confirm this hypothesis, it will be necessary to simultaneously record the activity of the distinct layers. Such a task will additionally require the use of cell-type-specific fluorescent markers with spectral properties complementary to those of the genetically encoded sensors. Furthermore, the causality of one network with respect to the activity of another could be further explored by combining optical monitoring of one layer with electrical^63^ and/or optogenetic stimulation^64–66^ of the other layer.

The observed effects of adjacent hippocampal cultures on the organization of cortical networks indicate an active interplay between the layers. Indeed, the assembly of 3D layered cultures with a contiguous interface represents an intriguing brain-on-chip model that can be used to study the establishment of cell-cell connections and communications between distinct neuronal populations over both long and short spatial ranges. Therefore, the proposed system could represent an effective model for investigating how modular network organizations emerge, for studying the roles of molecules released by specific nearby cells mimicking physiological or pathological conditions, and for studying the role of stimulus-evoked activity in physiologically relevant models such as the thalamocortical tissue interface.

Although the pace of progress in neuroscience constitutes a major stimulus for technological achievement, complex and/or expensive systems are still available in few skilled laboratories, and among them few research centers provide complementary knowledge that can be used to tackle specific neuroscientific questions^67^. For these reasons, we strongly believe that the proposed model of engineered *in vitro* brain tissue, together with the described platform for high-resolution and high-throughput functional screening assays, represents a simple and cost-accessible model for important advancements in the field of neuroscience, drug screening, and neural tissue engineering.

## Methods

### Ethical approval

All procedures involving experimental animals were approved by the Italian Ministry of Health and Animal Care (authorization ID 023, 15 April 2011). When performing the experiments, we minimized the number of sacrificed rats and the potential for nociceptor activation and pain-like sensation, and we respected the three R (replacement, reduction and refinement) principles, in accordance with the guidelines established by the European Community Council (Directive 2010/63/EU of 22 September 2010).

### Preparation of 3D neuronal cultures

Primary neurons were isolated from the brains of E18 Sprague-Dawley rats as previously described^68^. In particular, cortical neurons were obtained by 30 min incubation in 0.125% trypsin (25050014, Gibco, Thermo Fisher Scientific, MA USA 02451) + 0.01 mg/mL DNase (D5025-15KU, Sigma-Aldrich, Milan, Italy) at 37 °C. Hippocampal neurons were obtained by incubation in 0.25% trypsin-EDTA (25200056, Gibco, Thermo Fisher Scientific, MA USA 02451). After dissociation, the cells were washed in complete NB (Neurobasal Medium) (12348017, Gibco, Thermo Fisher Scientific, MA USA 02451) containing Glutamax (2 mM) (35050061, Gibco, Thermo Fisher Scientific, MA USA 02451) and penicillin (100 U/mL)/streptomycin (100 mg/mL) (17504044, Gibco, Thermo Fisher Scientific, MA USA 02451) and supplemented with 10% fetal bovine serum (FBS) (10270106, Gibco, Thermo Fisher Scientific, MA USA 02451) and centrifuged for 5 min at 335 × g. The pellet was resuspended in NB + 10% FBS. The cell suspension was filtered with a cell strainer (40 μm pore size, 352340, BD Falcon) and centrifuged for 7 min at 114 × g. Cells embedded in hydrogels were prepared as previously described^17^. In this work, rectangular parallelepiped-shaped (3 × 3 × 5 mm inner empty volume) 0.8% (w/v) agarose (A9539, Sigma-Aldrich, Milan, Italy) molds were prepared rather than the previously used donut-shaped molds. The cells were resuspended in filtered sterile 0.15% (w/v) sodium alginate (Pronova UP LVG, Novamatrix, Sandvika, Norway) at a density of 10^4^ cells/µL. The cell-alginate mixture (20 μL) was loaded into the agarose mold, which was surrounded by complete NB supplemented with CaCl_2_ (10 mM) (21108, Sigma-Aldrich, Milan, Italy). The slow radial diffusion of calcium ions through the agarose ensured homogeneous gelation of the resulting hydrogel. After 30 min, the gelation solution was exchanged for complete NB without additional CaCl_2_. Using these conditions, we obtained a hydrogel matrix with stiffness ranging from 10 to 100 Pascal measured with a rheometer under shear stress, as reported in our previous work; calibration of the stiffness of the matrix was obtained by adjusting the calcium and alginate concentrations (see Fig. 1 in ref. 17). Layered 3D co-cultures of cortical and hippocampal neurons were prepared in the same way using a two-step gelation procedure within the mold. For imaging of the layered structure at the interface, cortical and hippocampal neurons were transfected with the CAGGS_pIRES_tdTomato and AAV_syn_GFP plasmids, respectively, using the AMAXA^TM^ kit (V4XP-3024, Lonza, Basel, Switzerland). The transfected cells were incubated for 1 h at 37 °C; they were then centrifuged at 180 × g for 5 min, resuspended in 0.15% alginate and loaded into the agarose mold for gelation.

### Calcium imaging and viral infection of 3D neuronal cultures

Primary neurons were infected with the adeno-associated virus AAV_2.1_-EF1a-GCaMP6s as previously described^4^. Briefly, cell infection was performed at 10 DIVs by incubation with the virus (7160 transforming units/cell) in NB medium. The infected cell cultures started to express GCaMP6s at 17 DIVs. Calcium imaging was performed starting at 7 DIVs. To observe the calcium activity at time points earlier than 17 DIVs, the cells were labeled with the calcium indicator Fluo-4-AM (4.75 μM) (F14201, Molecular Probes, Thermo Fisher Scientific, MA USA 02451). 10 DIVs was chosen as the time for starting the incubation because it allowed good GCaMP6s expression without affecting the culture. As a calcium sensor, we chose GCaMP6s because it produced a higher SNR for detecting fluorescence fluctuation events either associated with one or ten action potentials (APs) compared to the other genetically engineered GCaMP proteins^24^.

Drug-control experiments were performed at 19–26 DIVs. In particular, tetrodotoxin (1 μM) (T8024, Sigma-Aldrich, Milan, Italy) was applied for 20 min, and bicuculline (30 μM) (285269, Sigma-Aldrich, Milan, Italy) was applied for 10 min; AP5 (20 μM) (A5282, Sigma-Aldrich, Milan, Italy) and DNQX (20 μM) (D0540, Sigma-Aldrich, Milan, Italy) were applied together for 20 min.

### Immunostaining and image analysis

Immunostaining was performed as previously described^17^. Primary antibodies were: guinea pig anti-VGLUT1 (135304, SYSY), rabbit anti-VGAT (131013, SYSY), and neuronal class III beta-tubulin antibody (clone TUJ1) (Covance, MMS-435P, Princeton, NJ, USA) diluted 1:250. Secondary antibodies were: Alexa Fluor 488 goat anti-guinea pig IgG (H + L) (A11073, Life Technologies, Carlsbad, CA, USA), and Alexa Fluor 568 goat anti-rabbit IgG (H + L) (A11036, Life Technologies, Carlsbad, CA, USA). All antibodies were diluted 1:500 except where otherwise specified. Nuclei were stained with Hoechst dye (3 μg/mL) (B2261, Sigma Aldrich, Milan, Italy). Images were acquired with a Leica SP8 confocal microscope and analyzed with ImageJ.

### Viability testing

3D cortical cultures were stained by incubation for 1 h at 37 °C with Hoechst dye (3 μg/mL) (B2261, Sigma Aldrich, Milan, Italy), which labels cell nuclei. Images were acquired with a Leica SP8 confocal microscope and analyzed with ImageJ. Apoptotic cells, which are characterized by pyknotic nuclei, were identified by their morphology and counted, as was the total number of cells. We counted approximately 140 ± 36 cell nuclei on z-stacks with a volume of 310 × 310 × 40 µm. We analyzed three distinct z-stacks for each condition (n = 3 hydrogels for each DIVs condition reported in Fig. 1e). Viability was calculated as the percentage of live cells divided by the total number of cells.

### Tunable extended-depth-of-field microscope

The optical layout was based on a custom-made wide-field inverted fluorescence microscope. The excitation light coming from a laser diode (DPSS-473-NL40, EKSMA Optics, Vilnius, Lithuania) passed through a laser speckle reducer (LSR3005, Optotune, Dietikon, Switzerland) to produce homogeneous field illumination; it was then collected with an L3 lens and reflected toward the objective (10x, 0.25 NA air objective, Olympus, Thorlabs, Munich, Germany) by a dichroic mirror DM (FF510-DI01, Semrock, Optoprim, Milan, Italy). The fluorescence emission light coming from the sample was separated from the excitation light through the DM and collected by a tube lens (ITL200, Thorlabs, Munich, Germany) oriented toward the detection optical path. The detection pathway consisted of a telescope in 4 f configuration between the tube lens and the CCD camera sensor (EM-CCD Andor Ixon DU897, Andor Technology plc, Belfast, UK), two convex lenses (L1 and L2) with equal focal lengths (AC254-100-A,Thorlabs, Munich, Germany), and a tunable lens (TL) placed in the common focal plane of L1 and L2. The TL (EL-10-30-C-VIS-LD, Optotune, Dietikon, Switzerland) focal length was controlled by an external voltage signal generated by an oscilloscope (PCSGU250, Velleman, Gavere, Belgium) and conditioned by a custom-made power driver. When the curvature of the liquid lens changed, the phase function of the telescope and the convergence of the detected signal at the CCD plane varied (solid and dashed lines in supplementary Fig. S2a). The composed telescope ensured that the magnification of the system remained constant while the axial position of the in-focus image plane projected on the CCD was varied, making it possible to perform inertia-free axial scanning of the sample. In front of the CCD, a fluorescence emission filter F1 (FF01-520/35, Semrock, Optoprim, Milan, Italy) rejected stray residual excitation light. To calibrate the axial shift of the sample in-focus plane with respect to the voltage driving signal of the TL, we prepared a microscope slide with sub-resolved fluorescent microspheres (Ø = 170 nm, Invitrogen Corp, Eugene, OR) dispersed on the surface; these were moved out of focus with a known axial shift through a piezoelectric translator and then refocused by changing the voltage on the TL. We repeated the axial shift for several steps to obtain the voltage (TL voltage signal) versus µm (axial displacement of the sample) relationship (supplementary Fig. S2b).

During experiments with neuronal networks, we used a D/A board (PCI-6529, National Instruments, Milan, Italy) and a custom-made LabVIEW software interface to acquire the external voltage signal sent to the TL and the exposure signal of each acquired image of the CCD to make it possible to associate the in-focus plane position with the corresponding image frame. To perform axial scanning, we drove the TL with a smooth sinusoidal voltage signal, thereby avoiding the resonant behavior of the TL and temporal oscillations of its focal length^69^ when high-frequency driving signals were present^69^.

Cells were maintained under the microscope at 35 °C using a Peltier device (QE1 resistive heating with a TC-324B heater controller, Warner Instruments, Holliston, MA 01745, USA). The pH and humidity of the neuronal cultures were controlled by aerating a custom-designed sleeve with humidified carbogen^70^.

### Wide-volume functional imaging protocol

With the TL set to a fixed in-focus position (constant voltage to TL), we chose an FOV of the 3D neuronal network. We then slowly scanned the sample in the axial direction by varying the voltage applied to the TL to visually inspect it and set the maximum and minimum voltage (V_max_ and V_min_ in supplementary Fig. S2c); in this way, we chose the portion of the sample volume to be monitored.

The velocity of the axial shift of the in-focus plane (V_focus-shift_) position between p_0_ and p_1_ (corresponding to V_p0_ and V_p1_, respectively) was controlled by varying the frequency of the TL sinusoidal driving signal (f_TL_) (see supplementary Fig. S2c). The frame rate acquisition of the CCD (f_CCD_) can be controlled independently to achieve a good signal-to-noise ratio. Therefore, we synchronized the CCD image acquisition with the axial shift of the in-focus plane to collect a z-stack of the 3D network and successively a t-stack of the extended depth-of- field (EDOF) images.

The z-stack of the 3D neuronal network was acquired by setting f_CCD_»f_TL_. The number of frames acquired in the z-stack (N_z-stack_) during the axial excursion of the sample from p_0_ to p_1_ was equal to N_z-stack_ = (f_CCD_/f_TL_)/2.

The dimension of the axial steps between consecutive frames of the z-stack was obtained by multiplying the TL voltage signal corresponding to each image by the constant calibration factor expressed in µm/volt (supplementary Fig. S2b). We set the f_CCD_ to 65 Hz, binning to 2 × 2, and the f_TL_ to 140 mHz; thus, we acquired 232 images for a complete excursion of the sample of approximately 300 µm from p_0_ to p_1_. The z-stack was processed with a custom-written algorithm in MATLAB to localize the cells within the scanned volume. As a first step, the standard deviation z-projection of the z-stack was obtained, and the non-homogeneous background in the projection image was estimated through a morphological opening operation with a disk of arbitrary size (smaller than the typical dimensions of the cell somata) and then subtracted. Successively, the projection image was binarized, and the ROIs were detected. Considering that the GCaMP6s indicator is mainly expressed in the cell soma, the fluorescence signal in the soma masks the signal in the neurites (see supplementary Videos S2–S4); thus, it already allows us to discard neuronal processes from the ROIs detection analysis. Furthermore, the image analysis algorithm is designed so that neuronal processes that express a comparable level of the GCaMP6s indicator can be discarded. In detail, after the binarization of the standard deviation projection, erosion and dilation of the binarized image is performed to discard thin/sharp components such as neuronal processes. The user can manually set the parameters for the image analysis (histogram threshold for the image binarization, putative minimal dimension, in pixela, of the ROIs and dimension of the erosion/dilatation element) through a GUI, which then shows the detected ROIs (typical parameter values: binarization threshold ≈0.3–0.5 applied to the normalized histogram of the intensities of the z-projection image; minimal ROIs dimension ≈2–5 pixels; erosion/dilatation element size ≈2–3 pixels; 1 pixel = 3.75 µm). Once the parameters have been set for a given optical magnification, pixel dimensions, and sensitivity of the detector used, they can be successfully applied to all recordings (with same acquisition parameters previously mentioned) to discard the neuronal processes and noise components. The axial position of the ROIs was obtained by calculating the mean intensity value of each ROI in all the frames of the z-stack (Fig. 2c). When more cells were aligned on the same axis, we detected more than one maximum on the intensity trace (supplementary Fig. S4). In this case, we assigned the cell location to the highest maximum. Although this represented a rough approximation, this condition was rare with the neuronal density of our 3D networks, and it occurred in only approximately 1–3% of the detected ROIs.

To increase the 3D localization accuracy of the cells, we collected several back-and-forth excursions of the sample between p_0_ and p_1_ and used these to compute the average intensity trace of each ROI. Each acquisition period of TL reads the sample two times (back and forth), and the acquisition frames are slightly shifted between the two readings (supplementary Fig. S3), thus allowing a further gain in localization accuracy. We determined the axial localization of the cells with a confidence interval of approximately 0.1 µm.

To also obtain the confidence interval for the transverse localization of the ROIs, we performed the following procedure. We randomly selected 500 of the total recorded frames and computed their standard deviation projection. We used the projection to determine the X and Y positions of the ROIs. By repeating this procedure 10 times, we obtained 10 copies of each ROI. In this way, we estimated confidence intervals of 0.22 µm and 0.21 µm for the X and Y positions, respectively.

The t-stack of the 3D neuronal network was acquired by setting f_TL_ »f_CCD_. In this case, we set f_TL_ to 140 Hz and maintained f_CCD_ at 65 Hz. Therefore, the entire axial portion of the sample was scanned two times back and forth within the time period of one image acquisition. In this way, we obtained a hardware-aided z-projection of the sample. We acquired a time lapse t-stack of the hardware projection images and used this to monitor the calcium fluctuations of the neurons. The fluorescence traces of the neurons were extracted from the t-stack by computing the mean fluorescence intensity value within the ROIs that were previously identified in the software-aided projection of the sample.

### Perona-Malik filter

The Perona-Malik filter produces a smoothed version of a signal by solving an evolutionary PDE up to a defined end time T. For increasing iterations T, a family of more simplified functions representing the original signal is computed as a scale-space representation.

The solution *u* of the original Perona-Malik filter is based on the following PDE:

1 $$\,\frac{{\rm{\delta }}{\rm{u}}}{{\rm{\delta }}{\rm{t}}}=\nabla \cdot ({\rm{g}}(|\nabla {\rm{u}}|)\nabla {\rm{u}})$$

where for each t, u(x,t) denotes a smoothed version of the original (scalar) signal u(x,0).

The so-called edge-stopping (or diffusivity) function *g* must be a smooth non-increasing function, with g(0)=1, g(s) ≥ 0 and *lim*_s→∞_ *g*(*s*) = 0 that depends on a positive parameter λ, called the contrast parameter. In this work, we consider the following form for the diffusivity function g:

2 $$g(x)=\{\begin{array}{c}\frac{1}{2}{(1-{(\frac{x}{{\rm{\lambda }}})}^{2})}^{2}\,if\,|x| < \lambda \\ 0\quad \quad \quad \quad \quad \quad \,if\,|x|\ge \lambda \end{array}$$

The choice of the edge-stopping function g and the contrast parameter λ determines the diffusion of the function itself, which is stopped or reduced when the norm of the gradient of the signal |∇u| exceeds the fixed λ. We observe that the diffusivity in the Perona-Malik model, or in similar approaches, depends locally on the modulus of the gradient of the function (signal). Here, we introduce a new diffusion coefficient that accounts for both the monotony of the signal and the presence of oscillations, which are typically due to noise. In other words, a high modulus of the gradient may lead to a small amount of diffusion if the function is also locally monotonic. Then, in our modified version of the Perona-Malik filter, we replace the argument of the function g, which regulates the diffusion of u, with a non-local term, making it possible to consider the behavior of the signal on some interval and not only pointwise. We remark that in our case, the signal is a one-dimensional signal and that x varies in a limited interval of the real line. First, we fix a length δ and compute, for each x, the local variation LV_δ_(u) = |u(x + δ) − u(x)| of the signal with respect to the subinterval [x, x + δ]. Furthermore, we compute the so-called total variation of the signal TV_δ_(u) on the same interval [x, x + δ] to have an estimate of the oscillatory component of the signal. The value of TV_δ_(u) is defined as the least upper bound of the variation $\sum _{{\rm{i}}=0}^{{{\rm{n}}}_{{\rm{p}}}-1}|{\rm{u}}({{\rm{x}}}_{{\rm{i}}+1})-{\rm{u}}({{\rm{x}}}_{{\rm{i}}})|$ of u over all the partitions ${{\mathscr{P}}}_{[{\rm{x}},{\rm{x}}+{\rm{\delta }}]}=\{{\rm{x}}={{\rm{x}}}_{0} < {{\rm{x}}}_{1} < \ldots  < {{\rm{x}}}_{{{\rm{n}}}_{p-1}} < {{\rm{x}}}_{{{\rm{n}}}_{p}}=x+{\rm{\delta }}\}$ of the interval [x, x + δ]. The new argument of the edge-stopping function is defined as the following ratio:

3 $$\frac{L{V}_{\delta }(u)}{T{V}_{\delta }(u)+\varepsilon }(x)=\frac{|u(x+\delta )-u(x)|}{su{p}_{{P}_{[x,x+\delta ]}}{\sum }_{i=0}^{{n}_{P}-1}|u({x}_{i+1})-u({x}_{i})|+\varepsilon }$$

where the positive parameter ε is a small parameter that is introduced to prevent division by zero.

Therefore, equation (1) of the Perona-Malik filter becomes

4 $$\frac{\delta u}{\delta t}={\nabla }\cdot (g(|\frac{L{V}_{\delta }(u)}{T{V}_{\delta }(u)+\varepsilon }|){\nabla }u)$$

In contrast to the classical Perona-Malik method, the parameters that regulate our filter do not depend on the derivative of the function *u*; they thus allow the preservation of subthreshold peaks or edges.

We used the following parameters: λ = 0.447, δ = 15 and T = 10. We set the parameters for the Perona-Malik filter by optimizing the number of true detected events while attempting to keep the number of false detected events low.

On the smoothed traces, we detected events when a series of conditions were fulfilled. In particular, (i) during the event onset, the fluorescence had to show an increase; (ii) an event had to produce an increase in the slope of the trace; (iii) after the event’s offset, the fluorescence had to decrease; and (iv) the time difference between the event’s onset and the event’s offset had to be within a certain time interval to prevent any late or false detection (i.e., random increases in the fluorescence due to general changes in the light intensity could be confounded with calcium transients). Given our recording conditions (SNR, acquisition rate, CCD sensitivity, pixel dimension, etc.), we used training data to set the parameters that we subsequently used for event detection: (i) the first derivative in a right interval of the onset overcomes a fixed positive threshold (10^−3^ if the trace obtained by averaging the fluorescence traces of all detected neurons does not present any calcium transient due to simultaneous activation of all the ROIs, 10^−2^ otherwise); (ii) the ΔF between the onset and the offset of an event overcomes a threshold defined as the standard deviation of the difference between the original and the smoothed trace; (iii) the first derivative in a right interval of the event offset is lower than a fixed negative threshold (−10^−4^); (iv) the time interval between the last time point after the onset with first derivative higher than a fixed threshold and the offset did not reach a fixed width (600 time points in case the trace obtained by averaging the fluorescence traces of all detected neurons showed onset events slower than 5 s, such as the hippocampal neurons in Fig. 4b; otherwise 300 time points). To compute the decay time of the detected ΔF/F0 peaks, we defined the half-decay time of the peak as the time required for the trace to fall to half its maximum value from the offset time point.

To create a benchmark for the performance of the modified PM filter, we simulated fluorescence traces with additive white Gaussian noise. The calcium transients were modeled as the impulse response of an autoregressive model^26^ of order 2 that simulated the rise and decay time constants of the GCaMP6s sensor. The underlying spikes were modeled as a homogeneous Poisson process with a firing rate of 0.05 Hz. The rise time and half-decay time constant were set to 0.5 s and 1 s, respectively^24^. We set the variance of the noise so as to have SNR = 9, similar to the experimental data. SNR is defined as the ratio of the maximum value of the signal to the standard deviation of the difference between the signal and a low-pass-filtered version of the signal. We further evaluated the performance of the filter as a function of the SNR by varying the variance of the noise so as to produce an SNR ranging between 8 and 11.

### Demixing algorithm

We adopted the demixing algorithm described in Paninski *et al*.^26^ that was used to demix the signal of neuronal cells overlapping in the axial direction as described in Yang *et al*.^21^. The code of the algorithm can be downloaded from the following link: https://github.com/epnev/ca_source_extraction. In more detail, we input the detected ROIs to the software and, using these ROIs as a starting point, estimated the calcium traces from the corresponding t-stack using the provided software. We then applied our algorithms to the extracted and demixed traces to detect the neural events.

### Neuronal network synchronicity and clustering

To quantify and compare the coordinated activities of the cell cultures, we measured the synchronous activity of the networks. We used the SPIKE-synchronization measure, which quantifies the degree of synchronicity within a neural network based on the relative number of quasi-simultaneous appearances of neural events^71^. Then, to identify neuronal populations with similar activity patterns, we applied a spike train community algorithm^72^ that self-determines the number of neuronal clusters based on the temporal discretization (i.e., bin size) used to compare the activities of the different cells. The time bin (varying from 0.01 s to 1 s) that yielded the highest modularity score^72^ was then used to cluster the calcium event trains.

### Neuronal network functional connectivity

The functional connectivity of the network was evaluated with the transfer entropy^73^ (TE) method. This method measures the amount of directed information between two processes and is computed as

5 $${\rm{TE}}({\rm{Y}}\to {\rm{X}})=\sum {\rm{p}}({{\rm{x}}}_{{\rm{n}}+1},{{\rm{x}}}_{{\rm{n}}}^{{\rm{m}}},{{\rm{y}}}_{{\rm{n}}}^{{\rm{m}}})\mathrm{log}\,\frac{{\rm{p}}({{\rm{x}}}_{{\rm{n}}+1}{|x}_{{\rm{n}}}^{{\rm{m}}},{{\rm{y}}}_{{\rm{n}}}^{{\rm{m}}})}{{\rm{p}}({{\rm{x}}}_{{\rm{n}}+1}{|x}_{{\rm{n}}}^{{\rm{m}}})}$$

where ${x}_{n}^{m}$ and ${y}_{n}^{m}$ are *m*-tuples of measurements at time steps $n,\,\ldots ,n-m$. All TE analysis were performed at a setting of m = 1 (approximately 15 milliseconds, with a frame rate of 65 Hz^74^).

Unlike the similarity measure used to cluster spiking activities, the TE allows the investigator to infer the directionality of the information flow since it is computed from current (n) and past (n − 1) values.

To perform a statistical assessment of the identified links, we create a null model that shuffles the neural events^75^ (n = 200) by maintaining the distribution of IEIs (inter-event intervals). For a given data set (X, Y), we compute the so-called Z-score as follows:

6 $$Z(TE)=\frac{TE-\mu (T{E}_{s})}{\sigma (T{E}_{s})}$$

where µ(TE_s_) is the mean value of a sample s under a null hypothesis of independence and σ(TE_s_) is the respective standard deviation. To obtain a sparse reconstruction of the network, only pairs with Z-scores higher than the 95^th^ percentile were considered significant and were included in the analysis.

Then, to corroborate the analysis performed on the clustering of the firing patterns, we analyzed the directionality of the functional links. In a polar coordinate reference system, we analyzed the azimuth and the polar angles (the angles formed with respect to the z-axis and in the x-y plane).

### Statistics

Statistical analysis was performed in MATLAB. When comparing two populations of data, the nonparametric two-sided Mann-Whitney test (p = 0.05) was used. For Fig. 1d, statistical analysis was performed in OriginPro8. Two-way ANOVA with Tukey’s post hoc test (p = 0.05) for multiple comparisons was used in Fig. 1d (top); the Kruskal-Wallis test (p = 0.05) was used in Fig. 1e.

### Data availability statement

All relevant data are included in the article and its supplementary information file. The MATLAB implementation of the Perona-Malik filter and the event detection algorithm can be found at https://github.com/moni90/modified-PM-filter and at the linked repositories therein.

## Electronic supplementary material

Supplemntary video S1

Supplemntary video S2

Supplemntary video S3

Supplemntary video S4

SupplementaryInformation
