# Supplementary material for: Fast wide-volume functional imaging of engineered in vitro brain tissues
Source: Sci Rep. 2017 Aug 17;7:8499. doi: 10.1038/s41598-017-08979-8 (PMC5561227; doi:10.1038/s41598-017-08979-8)
Supplement: Supplementary file 5 — SupplementaryInformation [file 41598_2017_8979_MOESM5_ESM.doc]

**Fast wide volume functional imaging of engineered *in vitro* brain tissues**

Palazzolo G.^1,*^, Moroni M. ^1,2,3,*^, Soloperto A. ^1^, Aletti G.^4^, Naldi G. ^4^, Vassalli M. ^5^, Nieus T. ^6,§,#^ and Difato F^1,§,#^.

^1^ Department of Neuroscience and Brain Technologies, Fondazione Istituto Italiano di Tecnologia, Genoa, Italy.

^2^ Center for Neuroscience and Cognitive Systems @UniTn, Istituto Italiano di Tecnologia, Rovereto, Italy

^3^ Center for Mind/Brain Sciences, University of Trento, Rovereto, Italy

^4^ Dipartimento di Matematica, Università degli studi di Milano, Milano, Italy

^5^ Institute of Biophysics, National Research Council of Italy, Genoa, Italy.

^6^ Department of Biomedical and Clinical Sciences "L. Sacco", Università degli Studi di Milano, Milano, Italy.

^*^ These authors contributed equally to this work

^§^ These authors supervised the study

^#^ Correspondence to [thierry.nieus@unimi.it; francesco.difato@iit.it]

**Supplementary Information**


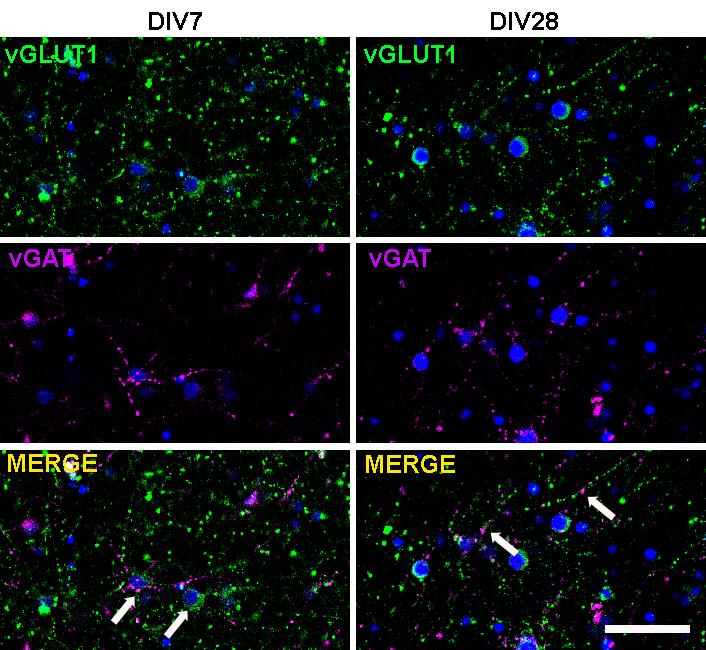


**Supplementary Figure S1. Localization of the synaptic markers vGLUT1 and vGAT in 3D cortical cultures over time.** Green: vGLUT1, magenta: vGAT, blue: nuclei, yellow: merge between green and magenta channels. Images were acquired with a Leica SP5 confocal microscope, 20X air objective. White arrows indicate the different pattern of localization of the two synaptic markers between DIV7 and DIV28. Bar is 50 µm.


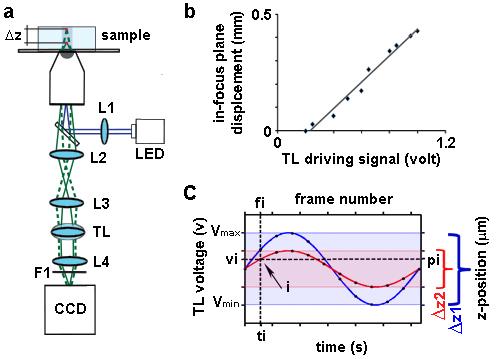


**Supplementary Figure S2. Scheme of the optical system and calibration of the extended depth of field imaging. a)** Optical layout of the system: L1, L2, L3, L4 = lens; TL = tunable lens; DM = dichroic mirror; F1= filter; CCD = charged coupled device; LED = Light Emitting Diode. The blue lines represent the optical path of the excitation light. The solid and dashed green lines illustrate the optical path of the emission fluorescence, with two distinct curvature of the TL lens. The curvature of the TL lens changes respect to the voltage signal applied to the TL lens, and the in-focus plane position is changed within the sample. The overall displacement of the in-focus plane in the sample is indicated as Δz; **b)** Calibration curve of the displacement of the in-focus plane acquired with the CCD versus the voltage applied to the TL lens; **c)** Determination of the extension of the microscope depth of field. The blue and red lines represent two distinct signals applied to the TL lens. The V_min_ and V_max_ represent the minimum and maximum voltage signal applied to the TL lens, which determine the extension of the microscope depth of field indicated as Δz1 and Δz2. A time point ti, on the voltage signal applied to the TL lens, determine the position pi within the sample, and the corresponding acquired frame number fi.


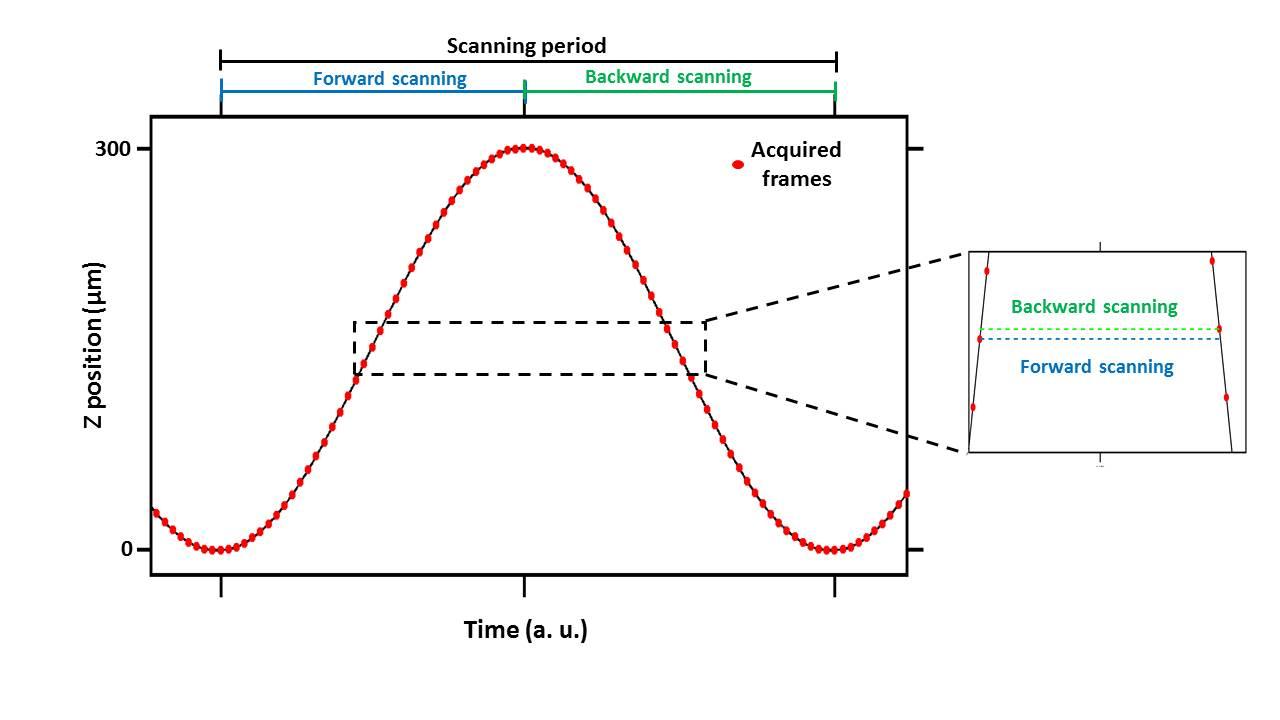


**Supplementary Figure S3. Scheme of the acquisition period during slow axial scan of the sample. a)** Each acquisition period of TL reads the sample two times (backward and forward scanning), and the axial positions in the sample during the frame acquisitions are slightly shifted between the two readings (indicated by the intersecting blue and green dashed lines in the inset). The shift amount (~ 250 nm) between the axial positions in the backward and forward scanning is maintained during repeated scanning periods of the sample in the axial direction.


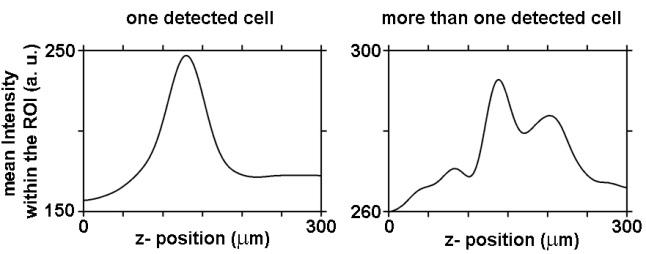


**Supplementary Figure S4.** Example of the obtained axial intensity profile computed in two detected ROIs of a 3D cortical culture at 26 DIVs. In case of a single cell within the identified ROI, the axial intensity profile present only one maximum. Otherwise, when more than one is present the axial intensity profile present more than one local maximum.


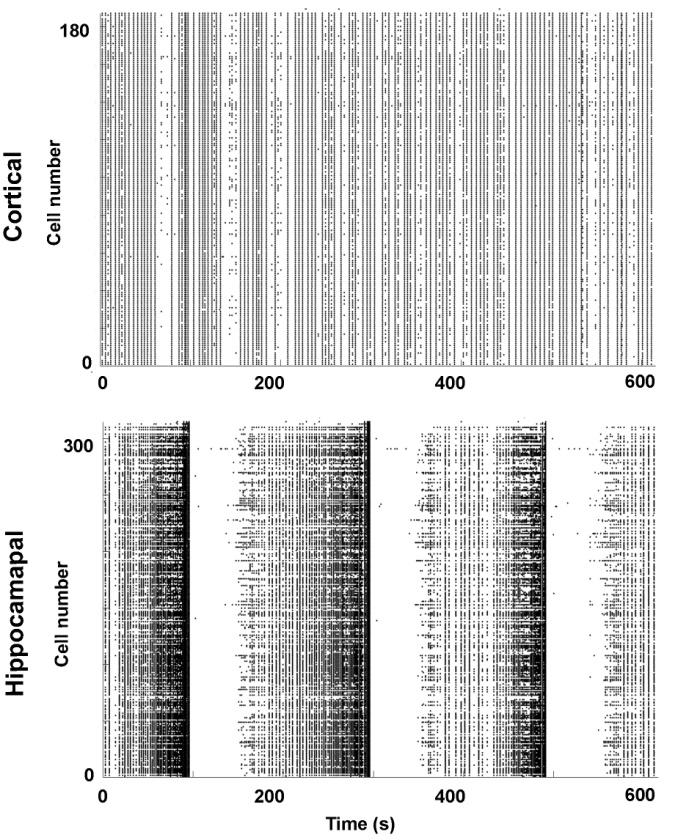


**Supplementary Figure S5. Raster plots of the detected calcium events of 3D neural networks.** We report two representative examples of raster plots of detected calcium events in cortical and hippocampal cultures at DIV26 *in vitro*.


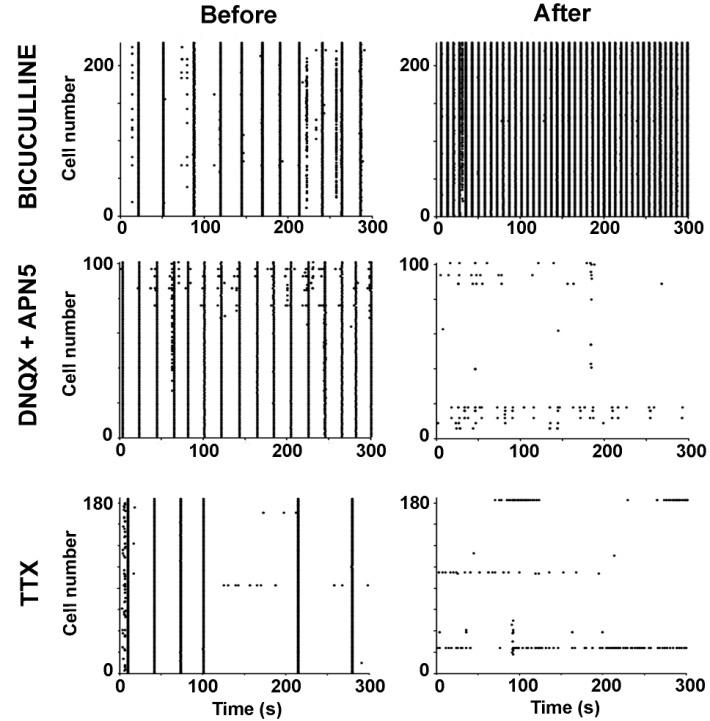


**Supplementary Figure S6. Raster plots of the detected calcium events of 3D neural networks before and after the addition of drugs.** Raster plots of the detected calcium events of 3D cortical neural networks, before and after the incubation with: the GABA_A_ receptor antagonist Bicuculline (30μM) (recording performed 10 min after incubation) at 19 DIVs, DNQX (20μM) and APN5 (20μM), respectively antagonists of NMDA and AMPA receptors (recording performed 20 min after incubation) at 19 DIVs, and TTX (1μM) that blocks the sodium channel conductance (recording performed 20 min after incubation) at 26 DIVs.

**Video legends**

**Supplementary Video1.** Movie of the 3D reconstruction of a cortical neuronal network (53 DIVs) by LAS AF software. Imaging was performed with an upright Leica SP5 confocal microscope. 25x Olympus water immersion objective.Volume view 350x300x300µm.

**SupplementaryVideo2.** Time lapse calcium imaging of a 3D neuronal network (26 DIVs) performed with a widefield microscope. Frame rate 20Hz, FOV 2.4x1.8 mm. Numbers indicate seconds. Bar is 350µm. Frame rate 20Hz, FOV 2.4x1.8 mm.

**SupplementaryVideo3.** Z-stack of the 3D neuronal network (24 DIVs) acquired with the TEDOF microscope working in Slow Scanning configuration. Frame rate 65 Hz.Z-scanning rate 0.14 Hz. Z-scanning Range: 0-300 µm. FOV 800x800µm. Number indicates µm. Bar is 150 µm.

**SupplementaryVideo4.** Time lapse calcium imaging of the 3D cortical neuronal network (24DIVs) of the supplementary video 3, performed with the TEDOF microscope working in Fast Scanning configuration. Frame rate 65 Hz. Z-scanning rate 140 Hz. Volume view of 800x800x300µm. Number indicates seconds. Bar is 150 µm.
